# UNC93B1 drives IL-6/STAT3-dependent tumor–macrophage crosstalk and epithelial–mesenchymal transition in breast cancer

**DOI:** 10.3389/fphar.2026.1887125

**Published:** 2026-08-06

**Authors:** Xinpeng Li, Hua Hao

**Affiliations:** Department of Pathology, Yangpu Hospital, School of Medicine, Tongji University, Shanghai, China

**Keywords:** epithelial–mesenchymal transition, IL-6/STAT3 signaling, lipid metabolism-related risk, macrophage polarization, single-cell sequencing, UNC93B1

## Abstract

**Background:**

Lipid metabolic reprogramming is increasingly recognized as a driver of breast cancer progression; however, how lipid metabolism-associated risk states are translated into immune remodeling and epithelial–mesenchymal transition (EMT) remains unclear. This study aimed to identify a lipid metabolism-related regulator that mediates tumor–macrophage crosstalk and to experimentally define its role in breast cancer progression.

**Methods:**

Lipid metabolism-related immune regulatory candidates were identified through integrated bulk transcriptomic, machine-learning, single-cell, and spatial transcriptomic analyses. UNC93B1 was prioritized for experimental validation using immunohistochemistry, gain- and loss-of-function assays, tumor cell–macrophage co-culture, ELISA, IL-6 neutralization, and STAT3 inhibition/rescue experiments.

**Results:**

Integrated multi-omics analysis prioritized UNC93B1 as a lipid metabolism-associated immune regulatory hub in breast cancer. UNC93B1 was associated with high lipid metabolism risk status, invasive tumor phenotypes, and stronger expression in aggressive molecular contexts, particularly HER2-enriched and triple-negative breast cancer samples. Single-cell and spatial transcriptomic analyses further linked UNC93B1 to myeloid/macrophage-enriched states, M2-like macrophage polarization, and Toll-like receptor-related immune niches. Experimentally, UNC93B1 knockdown suppressed breast cancer cell proliferation, migration, invasion, STAT3 activation, and EMT, whereas UNC93B1 overexpression produced the opposite effects. In tumor cell–macrophage co-culture systems, tumor cell-derived UNC93B1 enhanced IL-6 secretion, activated STAT3 signaling in macrophages, and promoted M2-like polarization. Reciprocally, macrophage-associated IL-6/STAT3 signaling reinforced EMT and invasive behavior in breast cancer cells. IL-6 neutralization and STAT3 inhibition markedly disrupted this paracrine feedback loop, confirming that UNC93B1 promotes breast cancer progression through an IL-6/STAT3-dependent tumor–macrophage crosstalk axis.

**Conclusion:**

This study identifies UNC93B1 as a macrophage-associated immune regulatory factor linked to lipid metabolism-related risk in breast cancer. Integrated multi-omics analyses and functional experiments demonstrate that UNC93B1 promotes IL-6/STAT3-dependent tumor–macrophage communication, thereby enhancing M2-like macrophage polarization, EMT activation, and invasive tumor behavior. These findings support UNC93B1 as a potential biomarker and intervention target in immunologically aggressive breast cancer. However, the direct role of UNC93B1 in lipid metabolic remodeling remains to be validated through lipidomic and metabolic flux analyses, and its tumor- and microenvironment-dependent effects require further confirmation in orthotopic or immune-competent *in vivo* breast cancer models.

## 1 Introduction

Breast cancer is not only a malignant tumor driven by genomic alterations but also a prototypical disease characterized by complex interactions between metabolic reprogramming and the immune microenvironment (Liang et al., 2023; Wan et al., 2025; Zhou et al., 2023). Despite advancements in early screening and molecularly targeted therapies, patients remain at risk of tumor progression and recurrence, processes closely linked to increased tumor cell plasticity and the dynamic remodeling of the tumor microenvironment (Baram et al., 2020; Liu S. et al., 2024). Recent research increasingly recognizes that metabolic reprogramming in tumor cells is not merely a passive result of tumorigenesis but also an active regulatory mechanism shaping the immune microenvironment (Dias et al., 2019; Gandhi and Das, 2019). Metabolic reprogramming can impact inflammatory cytokine secretion, immune cell function, and immune cell polarization, thereby fostering an immunosuppressive microenvironment (Liu Y. et al., 2024; Qiao et al., 2023). However, the mechanisms through which metabolic reprogramming interacts with inflammatory signaling pathways to sustain an immunosuppressive state in BRCA remain incompletely understood and need further systematic investigation.

Lipid metabolism has become a key element in this reprogramming process. It not only provides structural lipids and energy sources to tumor cells but also influences transcriptional networks, epigenetic changes, and intercellular communication through the production of various signaling intermediates (Feng and Kurokawa, 2020; Feng et al., 2025; Wan et al., 2025). In the tumor microenvironment, lipid availability and oxidative status directly impact immune cell behavior. Tumor-associated macrophages (TAMs), which are a major part of infiltrating immune cells, show significant metabolic flexibility (Hasan et al., 2022; Qiao et al., 2023). Fatty acid oxidation is generally linked to the M2 immunosuppressive phenotype, while the proinflammatory M1 phenotype depends heavily on glycolytic metabolism (Liang et al., 2023; Liu and Cao, 2023). Therefore, identifying crucial lipid metabolism-related genes, developing measurable risk models, and understanding their immune regulatory roles at single-cell and spatial levels are urgent steps to improve precision therapy in BRCA.

UNC93B1 was initially identified as an endoplasmic reticulum-localized regulator of Toll-like receptor (TLR) transport involved in initiating and maintaining inflammatory signaling (Lee et al., 2013; Pelka et al., 2018). Multi-omics data suggest that UNC93B1 shows abnormal expression across various tumor types and is closely linked to immune infiltration and activated inflammatory pathways, indicating it may have roles beyond traditional innate immune regulation (Tian et al., 2025; Yang et al., 2026). In the tumor microenvironment, where metabolic and inflammatory processes are highly interconnected, UNC93B1 could act as an upstream regulator connecting metabolic states to immune responses (Yang et al., 2026; Zhou et al., 2025). IL-6 triggers the IL-6R/gp130–Janus kinase–signal transducer and activator of transcription 3 (JAK–STAT3) pathway, creating a bidirectional inflammatory amplification loop between tumor cells and macrophages (Hu Z. et al., 2024; Huang et al., 2022; Johnson et al., 2018). Persistent activation of STAT3 stabilizes immunosuppressive macrophage polarization and increases tumor cell plasticity, forming a positive feedback loop that links metabolic reprogramming and immune remodeling, thereby driving malignant progression (Chang et al., 2013; Dong et al., 2023; Hu Y. et al., 2024).

In this study, we integrated bulk transcriptomic profiling, lipid metabolism-related risk modeling, single-cell RNA sequencing, spatial transcriptomics, and functional perturbation assays to define a lipid metabolism-associated immune regulatory program in BRCA. Unlike previous studies that primarily focused on isolated lipid metabolic signatures or individual inflammatory pathways, our study sought to identify candidate molecular mediators that connect lipid metabolism-related risk states with macrophage remodeling, spatial immune organization, and EMT-associated malignant progression. We identified UNC93B1 as a candidate immune-regulatory node associated with lipid metabolism-related risk, myeloid/macrophage-enriched transcriptional states, and IL-6/STAT3-dependent tumor–macrophage communication. These findings provide a multi-level framework for understanding how lipid metabolism-associated risk programs may be translated into immunosuppressive macrophage polarization and EMT-driven breast cancer progression.

## 2 Methods and materials

### 2.1 Data collection and processing

A total of 1,231 BRCA samples were acquired from The Cancer Genome Atlas (TCGA; https://portal.gdc.cancer.gov/), comprising 1,118 tumor samples and 113 normal tissue samples. Gene expression datasets (GSE20685 and GSE20711) were obtained from the Gene Expression Omnibus (GEO; https://www.ncbi.nlm.nih.gov/geo/). ScRNA-seq data were obtained from the GEO dataset GSE176078, and spatial transcriptomic data were obtained from dataset GSE290924. A total of 12,734 lipid metabolism-related genes were identified from the GeneCards (https://www.genecards.org/) using the keyword “lipid metabolism.”

Transcriptome data were converted to transcripts per million and log2-normalized, and batch effects were corrected using ComBat. Differentially expressed genes (DEGs) were identified using the thresholds |log fold change (logFC)| > 0.5 and false discovery rate (FDR) < 0.05. Single-cell data were subjected to quality control, normalization, dimensionality reduction, and clustering using the Seurat package (v5.3.1). Highly variable genes were selected, and uniform manifold approximation and projection (UMAP) was used for visualization. The Wilcoxon test was used to identify the marker genes for each cell subpopulation. Spatial transcriptomic data were aligned using the Space Ranger and subsequently imported into Seurat for normalization, dimensionality reduction, and clustering. Key gene expression distributions were visualized using spatial feature maps.

### 2.2 Differential expression analysis and weighted gene co-expression network analysis (WGCNA)

Differentially expressed genes between breast cancer and normal breast tissues were identified from the TCGA-BRCA cohort. Weighted gene co-expression network analysis was performed using the WGCNA package to build gene co-expression modules. The optimal soft-thresholding power was determined by assessing scale-free topology fit and mean connectivity across candidate powers. After constructing the network, the adjacency matrix was converted into a topological overlap matrix, and hierarchical clustering was carried out based on topological overlap dissimilarity. Co-expression modules were identified using dynamic tree cutting, followed by module–trait correlation analysis.

### 2.3 Gene Ontology (GO) and Kyoto Encyclopedia of Genes and Genomes (KEGG) enrichment analysis and protein–protein interaction (PPI) network construction

The intersection of the DEGs, WGCNA module-associated genes, and lipid metabolism-related genes retrieved from GeneCards was identified using a Venn diagram. GO analysis and KEGG pathway enrichment analysis for biological processes, cellular components, and molecular functions were performed using the R packages “clusterProfiler” and “org.Hs.eg.db”. The results were visualized using ggplot2 and ggpubr packages. GO and KEGG pathways were filtered using the adjusted p-value “qvalueCutoff” < 0.05, and only the top 10 enriched pathways were presented. PPI networks for intersecting genes were predicted using the Search Tool for the Retrieval of Interacting Genes database (https://string-db.org/), and the resulting network was visualized using Cytoscape software (version 3.9.1).

### 2.4 Machine learning for key gene lipid metabolism diagnostic models

By setting a random seed, the TCGA-BRCA dataset was divided into training (70%) and testing (30%) datasets. Six machine learning algorithms were applied for feature screening, including random forest (RF), support vector machine (SVM), gradient boosting machine (GBM), k-nearest neighbors (KNN), neural network, and least absolute shrinkage and selection operator (LASSO). The top 10 key genes identified by each algorithm were selected. Receiver operating characteristic (ROC) curves were generated from prediction probabilities, and AUC values were calculated using the pROC package to evaluate the classification performance of each model. The resulting key genes were combined with those identified by PPI analysis, and the intersection of these sets was used to select the critical genes. Finally, the pROC package was used to generate ROC curves, compute the area under the curve (AUC) for each gene, and assess the diagnostic value of individual key gene expression levels in BRCA.

### 2.5 Construction of a hub gene lipid metabolism risk prognostic model

Univariate Cox regression was initially used to identify the intersection genes associated with BRCA prognosis. Subsequently, the R package “glmnet” was applied for LASSO-Cox regression modeling. Finally, independent prognostic genes in BRCA were identified using multivariate Cox regression analysis. Patients in the TCGA cohort were stratified into low- and high-lipid metabolism-related risk (LMrisk) groups based on the median LMrisk value, and survival outcomes were analyzed using the Kaplan–Meier method. The LMrisk score was calculated using weighted LASSO-Cox coefficients derived from individual gene expression levels as follows:

$$\text{LMrisk}=\sum_{i=1}^{n}\beta_{i}\times \mathrm{Exp}i_{i}$$

where β_i is the regression coefficient and Expi_i is the corresponding gene expression level. The log-rank test was utilized to compare survival curves between groups. The R package “survivalROC” was used to determine the specificity and sensitivity of LMrisk in predicting 1-year, 2-year, and 3-year survival via ROC analysis and to calculate the AUC. A similar approach was applied to the GSE20685 and GSE20711 datasets to validate the applicability of LMrisk.

### 2.6 Construction of the LMrisk risk model nomogram and eXtreme gradient boosting (XGBoost) with SHapley additive exPlanations (SHAP) interpretation

Based on the results of multifactorial Cox regression analysis, LMrisk hub genes were incorporated into the nomogram model. The R package “rms” was employed to construct the nomogram and to evaluate the predictive accuracy of each LMrisk hub gene for patients with BRCA. An XGBoost prognostic model based on lipid metabolism-related genes was built using the XGBoost package in R. Model performance was assessed using the TCGA training set and multiple validation sets. The SHAP package was used to interpret the XGBoost model and generate SHAP summary and individual plots. This analysis quantified the contribution of each gene to the predictive outcomes of the model and confirmed the key prognostic role of hub genes.

### 2.7 Macrophage subpopulation re-segmentation

After quality control and clustering of the entire single-cell dataset, the macrophage populations were selected for secondary analysis based on established marker genes. Upon extracting the macrophage subpopulations, the Seurat package v5.3.1 was employed for renormalization, identification of highly variable genes, and principal component analysis (PCA) for dimensionality reduction. Based on the top 20 principal components, re-clustering was performed using the Louvain algorithm (FindClusters, resolution = 0.5) to characterize potential functional heterogeneity within macrophages. Subpopulation marker genes were identified using the Wilcoxon rank-sum test (FindAllMarkers, adjusted p < 0.05, |log_2_FC| > 0.25) and subsequently functionally annotated in conjunction with the literature-reported M1/M2 signature genes.

### 2.8 Pseudotime analysis and cell communication

Macrophage development and polarization trajectories were reconstructed using the Monocle2 package (v2.26.0) for pseudotime analysis. Developmental trajectories were constructed based on the highly variable and DEGs. The DDRTree dimensionality reduction algorithm was employed to visualize pseudotime distributions and infer the dynamic trends across subpopulations along their developmental pathways. Intercellular communication was subsequently inferred using the CellChat package (v2.1.2) to identify signaling interactions between macrophages and other immune cells or tumor cells via the human ligand-receptor database CellChatDB. Key communication axes and signaling networks were identified by calculating the information flow intensity and signaling pathway enrichment scores. The visualization functions netVisual_circle and netAnalysis_contribution were used to display the overall communication patterns and pathway contributions of selected key pathways.

### 2.9 Spatial transcriptomics analysis

After normalizing and filtering highly variable genes in the GSE290924 spatial transcriptomics dataset using Seurat (v5.3.1), dimensionality reduction and clustering were conducted using PCA and UMAP. M2-polarized regions were defined based on the M2 macrophage hallmark gene MRC1, and the spatial distribution patterns of the relevant gene modules were subsequently extracted. Spatial feature plots were used to visualize the spatially resolved expression of UNC93B1 and to conduct spatial colocalization analysis with M2 macrophage regions, thereby evaluating its spatial association with immunosuppressive macrophages within the tumor microenvironment.

### 2.10 Quantitative real-time fluorescent polymerase chain reaction (PCR) and western blotting

Total RNA was extracted from the various cell lines using the TRIzol reagent (Invitrogen, Thermo Fisher Scientific, USA) and subsequently reverse-transcribed into complementary DNA (cDNA) using a commercial kit (Takara, Tokyo, Japan). Quantitative reverse transcription PCR (qRT-PCR) was conducted using gene-specific primers. Primer sequences are provided in the Supplementary Materials (Supplementary Table S1).

For protein extraction, cell pellets collected after trypsinization were lysed in radioimmunoprecipitation (RIPA) buffer (Servicebio, China) supplemented with protease (1:100, Beyotime, China) and phosphatase inhibitors. After bicinchoninic acid assay (BCA)-based protein quantification, the samples were mixed with 2X/4X loading buffer and boiled for 5 min. Proteins were separated by sodium dodecyl sulfate-polyacrylamide gel electrophoresis (SDS-PAGE) and transferred to polyvinylidene fluoride (PVDF) membranes. After membrane blocking, the membrances were incubated with primary antibody overnight at 4 °C. The primary antibodies used included UNC93B1 (1:1000; Proteintech, 28359-1-AP), E-cadherin (1:1000; Proteintech, 20874-1-AP), N-cadherin (1:1000; Proteintech, 22018-1-AP), Vimentin (1:1000; Proteintech, 10366-1-AP), STAT3 (1:1000; Proteintech, 60199-1-Ig), p-STAT3 (1:1000; Proteintech, 60479-1-Ig), and β-actin (1:10,000; Proteintech, 66009-1-Ig). After three 10-min washes with Tris-buffered saline with Tween 20 (TBST), the membranes were incubated with the corresponding secondary antibodies. After three additional TBST washes, the membranes were developed for signal detection.

### 2.11 Immunohistochemistry (IHC)

The studies involving humans and mice were approved by the Ethics Committee of Yangpu Hospital Affiliated with Tongji University (Shanghai Yangpu District Central Hospital) and were in accordance with the Declaration of Helsinki. Approval code: LL-2021-WSJ-010 and 2023-SCI-013.

For the xenograft tumor model, 10 nude mice were inoculated with MCF-7 cells into the bilateral axillary regions. On day 21 after inoculation, the mice were euthanized and necropsied. The xenograft tumor tissues and adjacent non-tumorous peritumoral tissues surrounding the tumor margins were carefully dissected separately. For morphological evaluation, tissue sections were stained with hematoxylin and eosin (H&E). For immunohistochemical analysis, UNC93B1 protein expression in tumor tissues and adjacent non-tumorous peritumoral tissues was detected using an anti-UNC93B1 antibody.

Formalin-fixed, paraffin-embedded human breast cancer tissues and mouse xenograft tissues were sectioned into 4-μm-thick serial sections. Sections were dewaxed in xylene, dehydrated through graded ethanol, and rehydrated prior to antigen retrieval by autoclaving for 30 min. The sections were subsequently incubated with 3% hydrogen peroxide for 20 min to quench endogenous peroxidase activity. Nonspecific binding was blocked by incubating the sections with 5% bovine serum albumin at room temperature for 40 min. The sections were then incubated overnight at 4 °C with anti-UNC93B1 primary antibody (1:200; Proteintech, 28359-1-AP). The following day, the sections were washed, incubated with the corresponding secondary antibody, stained with a 3,3′-diaminobenzidine (DAB) chromogen kit, counterstained with hematoxylin, dehydrated, cleared, and mounted for microscopic observation.

### 2.12 Cell culture and transfection

The human BRCA cell lines MCF-7 and MDA-MB-231 and the mouse BRCA cell line 4T1 were purchased from the Shanghai Institute of Biochemistry and Cell Biology, Chinese Academy of Sciences. All cells were maintained in their respective growth medium supplemented with 10% fetal bovine serum (FBS; Servicebio, China) at 37 °C in a humidified incubator with 5% CO_2_. THP-1 cells were cultured in RPMI-1640 medium (Servicebio, China) supplemented with 10% FBS and 0.05 mM β-mercaptoethanol.

BRCA cells were transfected using Lipo8000™ transfection reagent (Beyotime, China). UNC93B1-specific siRNA was synthesized by GeneChem (China), and UNC93B1 overexpression and empty control plasmids were obtained from Nacheer (China). The siRNA sequences and plasmid information are provided in Supplementary Tables S2, S3, respectively. RNA was extracted 24 h post-transfection, and proteins were harvested 48 h post-transfection for subsequent functional assays. All experiments were independently replicated at least three times.

### 2.13 Cell Counting Kit-8 (CCK-8) assay

Cell proliferation was assessed using the Cell Counting Kit-8 (CCK-8; Dojindo, Japan). Treated cells were seeded in 96-well plates at a density of approximately 1 × 10^4^ cells per well in 100 µL culture medium and cultured for 12, 24, 48, 72, or 96 h according to the experimental design. At each indicated time point, 10 µL of CCK-8 reagent was added to each well, corresponding to a 10% reagent-to-medium ratio according to the manufacturer’s protocol. After incubation at 37 °C for 1 h, absorbance was measured at 450 nm using a microplate reader. Cell viability was calculated from the relative absorbance values and normalized to the corresponding control group. All experiments were independently repeated at least three times.

### 2.14 Scratch assay and transwell migration/invasion

Transfected cells were seeded into 6-well plates to form confluent monolayers and incubated until reaching confluence (90%–100%). A uniform vertical scratch was made across the cell monolayer using a sterile 100-μL pipette tip, and detached cells were gently removed by washing twice with phosphate buffered saline (PBS). Subsequently, low-serum medium (2% FBS) was added, and plates were incubated at 37 °C. The same field of view was photographed using identical microscope settings at 0, 24, and 48 h post-scratching. The migration rate was calculated using the following formula: migration rate (%) = (initial scratch area – scratch area at the specific time point)/initial scratch area × 100%. Each group included at least three replicate fields of view, and the experiment was repeated thrice.

Transwell chambers (8 μm, Labselect, China) compatible with 6-well plates were used for migration and invasion assays. For the migration assay, 1500 μL of a cell suspension (5 × 10^5^ cells/well, 2% FBS) was added to the upper chamber, and 2000 μL of complete medium containing 20% FBS was placed in the lower chamber to serve as a chemotactic stimulus. Plates were incubated at 37 °C for 16–24 h (adjusted according to cell line). In the invasion assay, the upper chamber membrane was pre-incubated with pre-thawed Matrigel (diluted according to the manufacturer’s instructions, coated, and solidified at 37 °C). The remaining steps were identical to those of the migration assay, with the exception that incubation time was extended to 48 h. Cells were fixed with 4% paraformaldehyde for 20 min, washed twice with PBS, stained with crystal violet for 20 min, rinsed with PBS, and air-dried. Following incubation, non-migrated or non-invading cells from the upper chamber were removed with a cotton swab. Cells that had traversed to the lower surface were enumerated under a microscope across at least five random fields of view or photographed and quantified using ImageJ. Results are expressed as the mean number of migrating cells, with all experiments performed in triplicate (n ≥ 3).

### 2.15 Macrophage polarization

THP-1 monocytes were counted, and cell density was adjusted to 5 × 10^5^ cells/well. The cells were differentiated into M0 macrophage-like cells by treatment with 200 ng/mL phorbol 12-myristate 13-acetate (PMA) for 36 h. Following differentiation, PMA-treated THP-1 cells were gently washed twice using PBS to remove residual PMA and subsequently cultured in complete medium for 24 h. Thereafter, 2 mL of the cell suspension was seeded into the lower chamber of a Transwell system for indirect co-culture. Transfected tumor cells were resuspended and adjusted to a density of 2.5 × 10^5^ cells/well, and a uniform 1.5 mL volume was added to the upper chamber of the co-culture system. Following cell adhesion, the upper chamber was transferred, and the cells were co-cultured with M0 macrophages for 48 h. Total RNA was extracted from THP-1 macrophages to assess expression of M1 and M2 macrophage markers.

### 2.16 Enzyme-linked immunosorbent assay(ELISA) assay

The concentration of IL-6 in the cell supernatants was determined using a double-antibody sandwich ELISA format with a mouse IL-6 ELISA kit (LiankeBio, China) according to the manufacturer’s instructions. Standard and sample solutions were added to a 96-well plate pre-coated with capture antibodies and incubated. After washing, biotin-labeled antibodies and horseradish peroxidase (HRP) conjugates were sequentially added. Following substrate-based color development, absorbance was measured at 450 nm using a microplate reader. IL-6 concentrations were interpolated from a standard curve. All experiments were independently replicated at least thrice.

### 2.17 Acquisition of conditioned medium and signal transducer and activator of transcription 3 (STAT3) inhibition and rescue experiments

4T1 mouse mammary carcinoma cells at the logarithmic growth phase were seeded into 6-well plates. Transfection was performed when the cells had reached 70%–80% confluence. The experimental group was transfected with the UNC93B1 overexpression plasmid, whereas the control group was transfected with an empty vector. After 24 h of transfection, the cells were transferred to serum-free medium and maintained for an additional 24 h. The supernatants were collected and centrifuged at 1,000 × g for 10 min to remove cellular debris, thereby yielding conditioned medium (CM) for subsequent macrophage treatment. For IL-6 neutralization, the conditioned medium was supplemented with an anti-mouse IL-6 neutralizing antibody (10 ng/mL; R&D Systems, Cat. No. MAB406) before being applied to the recipient cells.

RAW264.7 macrophages were pretreated with the 50 μM STAT3 inhibitor S3I-201 (Beyotime, China) or an equal volume of dimethyl sulfoxide (DMSO) for 2 h to suppress endogenous STAT3 signaling. Cells were then transferred to a medium containing 2% FBS and supplemented with CM and cultured for an additional 24 h. After treatment, the cells were harvested, and total protein was extracted for downstream analysis. STAT3 phosphorylation levels were assessed by Western blot to confirm the inhibition efficiency.

Using co-culture inserts (0.4 μm; Nest, China), RAW264.7 cells seeded in the upper chamber were pretreated with 50 μM S3I-201 for 2 h, followed by incubation with recombinant mouse IL-6 protein (10 ng/mL, Yeasen, China) for an additional 24 h. Subsequently, cells were harvested, and Western blot analysis was performed to evaluate the expression levels of phosphorylated (p)-STAT3 and proteins associated with EMT, thereby investigating whether IL-6 treatment could counteract the biological changes induced by STAT3 inhibition.

### 2.18 Data statistics

All experiments were repeated at least thrice. Data are presented as mean ± standard deviation (SD). Differences between groups were evaluated using Student’s t-test for pairwise comparisons or one-way analysis of variance (ANOVA) for multiple group comparisons. All statistical analyses were performed using GraphPad Prism software (version 10.1.2). Statistical significance thresholds are denoted as follows: *p < 0.05, **p < 0.01, ***p < 0.001, and ****p < 0.0001.

## 3 Results

### 3.1 Transcriptional remodeling and lipid metabolism-focused co-expression network identifies a BRCA-specific candidate gene landscape

To characterize transcriptional remodeling in breast cancer and identify lipid metabolism-related candidates for downstream modeling, we integrated differential expression analysis, WGCNA, and lipid metabolism gene annotation using the TCGA-BRCA cohort. The distributions of adjusted *P* values and dispersion estimates supported the reliability of the differential expression analysis, indicating broad transcriptional differences between BRCA and normal breast tissues (Supplementary Figures S1A,B).

**FIGURE 1:**
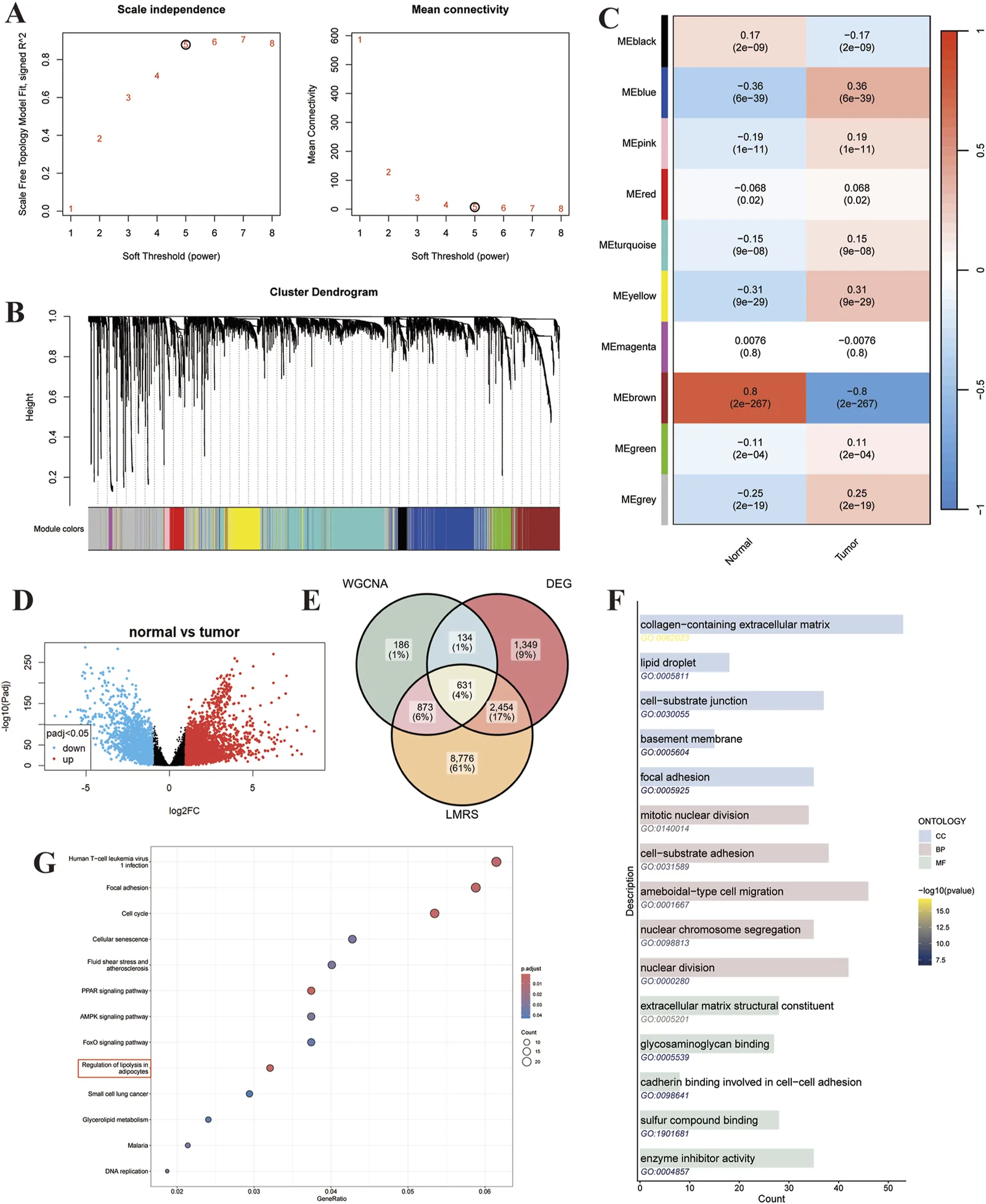
Integrated transcriptomic and co-expression network analyses identify lipid metabolism-related candidate genes in BRCA. **(A)** Determination of the optimal soft-thresholding power for WGCNA based on scale-free topology fit and mean connectivity. **(B)** Cluster dendrogram illustrating gene co-expression modules identified by WGCNA. **(C)** Module–trait correlation heatmap displaying associations between co-expression modules and BRCA or normal phenotypes. **(D)** Volcano plot illustrating differentially expressed genes between BRCA and normal breast tissues. **(E)** Venn diagram depicting the intersection of WGCNA module genes, differentially expressed genes, and GeneCards-annotated lipid metabolism-related genes. **(F)** GO enrichment analysis of candidate genes, including biological process, cellular component, and molecular function categories. **(G)** KEGG pathway enrichment analysis revealing lipid metabolism- and cancer-related pathways enriched among candidate genes.

A total of 4,567 differentially expressed genes were identified, including 2,863 upregulated and 1,704 downregulated genes, indicating extensive transcriptional reprogramming in BRCA (Figure 1D). Subsequently, Weighted Gene Co-Expression Network Analysis was conducted to elucidate coordinated gene expression patterns. Based on scale-free topology fitting and mean connectivity, an optimal soft-thresholding power of β = 6 was selected (Figure 1A). Hierarchical clustering revealed the presence of ten co-expression modules, reflecting that BRCA-related transcriptional modifications are organized into distinct modular structures (Figure 1B). The topological overlap matrix further demonstrated clear module boundaries and strong intramodule connectivity, thereby supporting the robustness of the constructed network (Supplementary Figure S1C).

Module–trait correlation analysis indicated that the blue and yellow modules are strongly linked to the BRCA phenotype (Figure 1C). Functional enrichment analysis showed that genes in these modules are involved not only in cancer-related processes such as extracellular matrix remodeling, focal adhesion, cell–substrate adhesion, nuclear division, and chromosome segregation, but also in lipid metabolism-related pathways, including lipid droplet formation, glycerolipid metabolism, regulation of lipolysis, AMPK signaling, and PPAR signaling (Figures 1F,G). These results suggest that lipid metabolic remodeling in BRCA is closely connected with cell proliferation, matrix restructuring, and metabolic signaling.

Finally, we intersected genes from the key WGCNA modules, differentially expressed genes, and GeneCards-annotated lipid metabolism-related genes, yielding 631 BRCA-associated lipid metabolism-related candidate genes (Figure 1E). Network visualization further demonstrated that these candidates formed a highly connected regulatory network, with several proliferation- and metabolism-associated genes occupying central positions (Supplementary Figure S1D). Collectively, these results establish a BRCA-specific lipid metabolism-related candidate gene landscape and provide a robust foundation for subsequent machine-learning-based feature selection, risk model construction, and mechanistic validation.

### 3.2 Machine learning establishes diagnostic signatures related to lipid metabolism in BRCA

**FIGURE 2:**
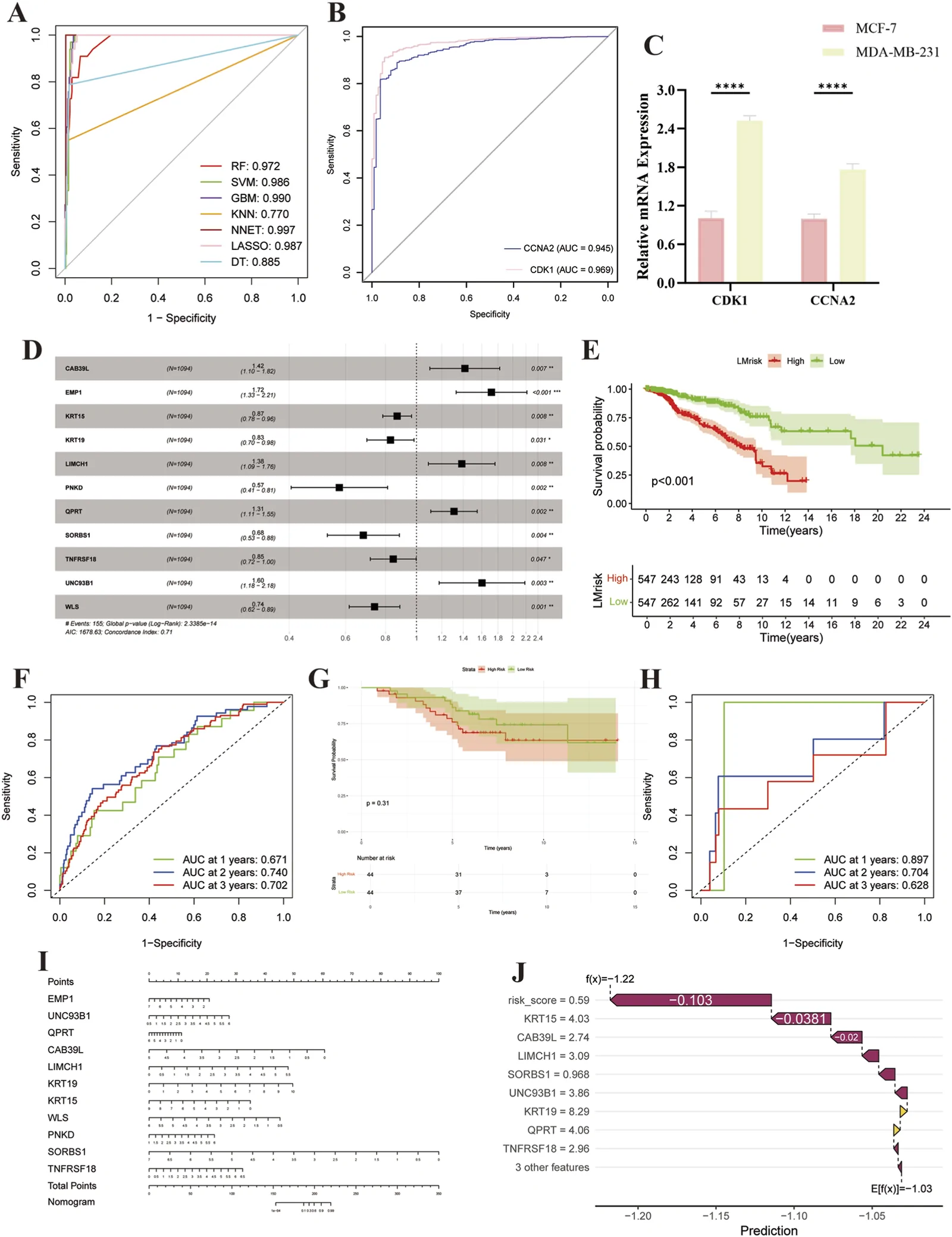
Construction, validation, and interpretation of lipid metabolism-related diagnostic and prognostic models in BRCA. **(A)** ROC curves comparing the diagnostic performance of multiple machine-learning models based on lipid metabolism-related genes. **(B)** ROC curves showing the diagnostic performance of the representative hub genes CDK1 and CCNA2. **(C)** qRT-PCR validation of CDK1 and CCNA2 expression in MCF-7 and MDA-MB-231 cells. **(D)** Forest plot of the final multivariate Cox regression model used to construct the 11-gene LMrisk signature. **(E)** Kaplan–Meier survival analysis comparing overall survival between high- and low-LMrisk groups in the TCGA-BRCA cohort. **(F)** Time-dependent ROC curves evaluating the predictive performance of the LMrisk model at 1, 2, and 3 years. **(G)** Kaplan–Meier survival analysis of the LMrisk model in the external validation cohort. **(H)** Time-dependent ROC curves assessing the performance of the LMrisk model in the external validation cohort. **(I)** Nomogram integrating the 11 LMrisk genes for individualized risk estimation. **(J)** SHAP waterfall plot illustrating feature-level contributions to an individual prediction. Statistical significance is indicated in the corresponding panels.

To translate the 631 BRCA-associated lipid metabolism-related candidates into diagnostic biomarkers, we established a multi-algorithm machine-learning framework. Seven classifiers, including RF, SVM, GBM, KNN, NNET, LASSO, and decision tree models, were systematically compared. Most models achieved strong discriminatory performance, particularly NNET, GBM, LASSO, SVM, and RF, with AUC values of 0.997, 0.990, 0.987, 0.986, and 0.972, respectively, indicating that lipid metabolism-related transcriptional features robustly distinguish BRCA from normal breast tissues (Figure 2A). The reverse cumulative distribution of residuals further showed that most algorithms maintained low residual errors, supporting the stability of the diagnostic framework (Supplementary Figure S1F).

To prioritize diagnostic genes with consistent predictive contribution, we integrated feature-importance rankings across machine-learning models. The permutation-based importance analysis identified several recurrent high-weight genes across classifiers, suggesting that the diagnostic signal was driven by a convergent set of lipid metabolism-related transcriptional features rather than by a single algorithm-specific pattern (Supplementary Figure S1E). Among these candidates, CDK1 and CCNA2 emerged as representative diagnostic genes. ROC analysis confirmed their strong individual discriminatory ability, with AUC values of 0.969 for CDK1 and 0.945 for CCNA2 (Figure 2B). Consistently, qRT-PCR validation showed significantly higher expression of both genes in MDA-MB-231 cells than in MCF-7 cells, supporting their association with a more aggressive BRCA phenotype (Figure 2C). Collectively, these results establish a robust lipid metabolism-based diagnostic signature and provide a rationale for extending this gene set toward prognostic modeling.

### 3.3 Construction and validation of an LMrisk model for prognostic stratification

Subsequently, we examined whether genes associated with lipid metabolism could encapsulate survival heterogeneity in BRCA. Univariate Cox regression analysis was initially employed to identify genes linked to survival within the realm of lipid metabolism. This was followed by LASSO regression to mitigate dimensionality and regulate model complexity (Supplementary Figure S2A–C). Ultimately, multivariate Cox regression facilitated the development of an 11-gene LMrisk model, comprising WLS, UNC93B1, TNFRSF18, SORBS1, QPRT, PNKD, LIMCH1, KRT19, KRT15, EMP1, and CAB39L (Figure 2D). The resultant model demonstrated considerable prognostic significance, with a global log-rank P value of 2.3385 × 10^−14^, an AIC of 1678.63, and a concordance index of 0.71.

Patients were subsequently categorized into high- and low-LMrisk groups based on the calculated risk score. The high-LMrisk group exhibited significantly poorer overall survival compared to the low-LMrisk group, indicating that the model effectively captured lipid metabolism-associated prognostic heterogeneity in BRCA (Figure 2E). Time-dependent ROC analysis further demonstrated predictive performance at 1, 2, and 3 years, with AUC values of 0.671, 0.740, and 0.702, respectively (Figure 2F). Consistently, analyses of risk score distribution, survival status, and heatmaps indicated that higher LMrisk scores were associated with increased mortality and distinct expression patterns of the 11 model genes (Supplementary Figure S2D–F).

To evaluate the generalizability of the model, the LMrisk signature was further assessed within an independent validation cohort. Although Kaplan–Meier analysis indicated a survival trend rather than definitive statistical separation, patients classified as high-LMrisk consistently demonstrated a poorer survival propensity. Time-dependent ROC curves preserved predictive information, especially for short-term prognostication, with a 1-year AUC of 0.897 in one validation cohort, and AUC values of 0.721, 0.599, and 0.616 at 1, 2, and 3 years respectively in another validation analysis (Figures 2G,H; Supplementary Figures S2G–J). These results imply that the LMrisk model effectively captures clinically pertinent metabolic risk signals across diverse datasets. Nonetheless, the relatively limited long-term predictive accuracy observed during external validation underscores the necessity for larger multicenter cohorts and prospective datasets to further refine and enhance the robustness and generalizability of this prognostic model.

### 3.4 Reinforced validation and interpretable modeling support the clinical relevance of LMrisk

To enhance the translational utility of the LMrisk model, we constructed a nomogram integrating the 11 model genes for individualized risk estimation (Figure 2I). The nomogram demonstrated excellent calibration and discrimination, with a C-index of 0.998 (95% CI: 0.996–0.999), indicating a strong concordance between predicted and observed outcomes (Supplementary Figure S3A). Compared to individual genes, the integrated nomogram exhibited superior predictive performance, with an AUC of 0.998, surpassing single markers such as SORBS1 (AUC = 0.965), EMP1 (AUC = 0.911), KRT19 (AUC = 0.848), and UNC93B1 (AUC = 0.815) (Supplementary Figure S3B). These findings suggest that the combined lipid metabolism-related gene signature offers greater predictive value than any single component alone.

To further investigate the robustness and interpretability of the model, validation using XGBoost and SHAP analysis were conducted. XGBoost achieved Area Under the Curve (AUC) values of 0.800 in the training dataset and 0.883 in the validation dataset, thereby supporting the reproducibility of the risk architecture across different modeling strategies (Supplementary Figure S3C,D). SHAP analysis identified the overall risk_score as the primary contributor to model prediction, followed by genes including CAB39L, LIMCH1, KRT15, KRT19, UNC93B1, SORBS1, TNFRSF18, EMP1, QPRT, PNKD, and WLS (Supplementary Figure S3E,F). The SHAP waterfall plot further illustrated how individual gene features cumulatively influenced patient-level predictions, providing a transparent explanation for the model-derived risk estimates (Figure 2J). Collectively, these validation analyses support the utility of LMrisk as a biologically grounded, interpretable, and clinically informative tool for lipid metabolism-based prognostic stratification in BRCA.

### 3.5 Single-cell transcriptomics identifies UNC93B1 as a myeloid-enriched component of the LMrisk signature

**FIGURE 3:**
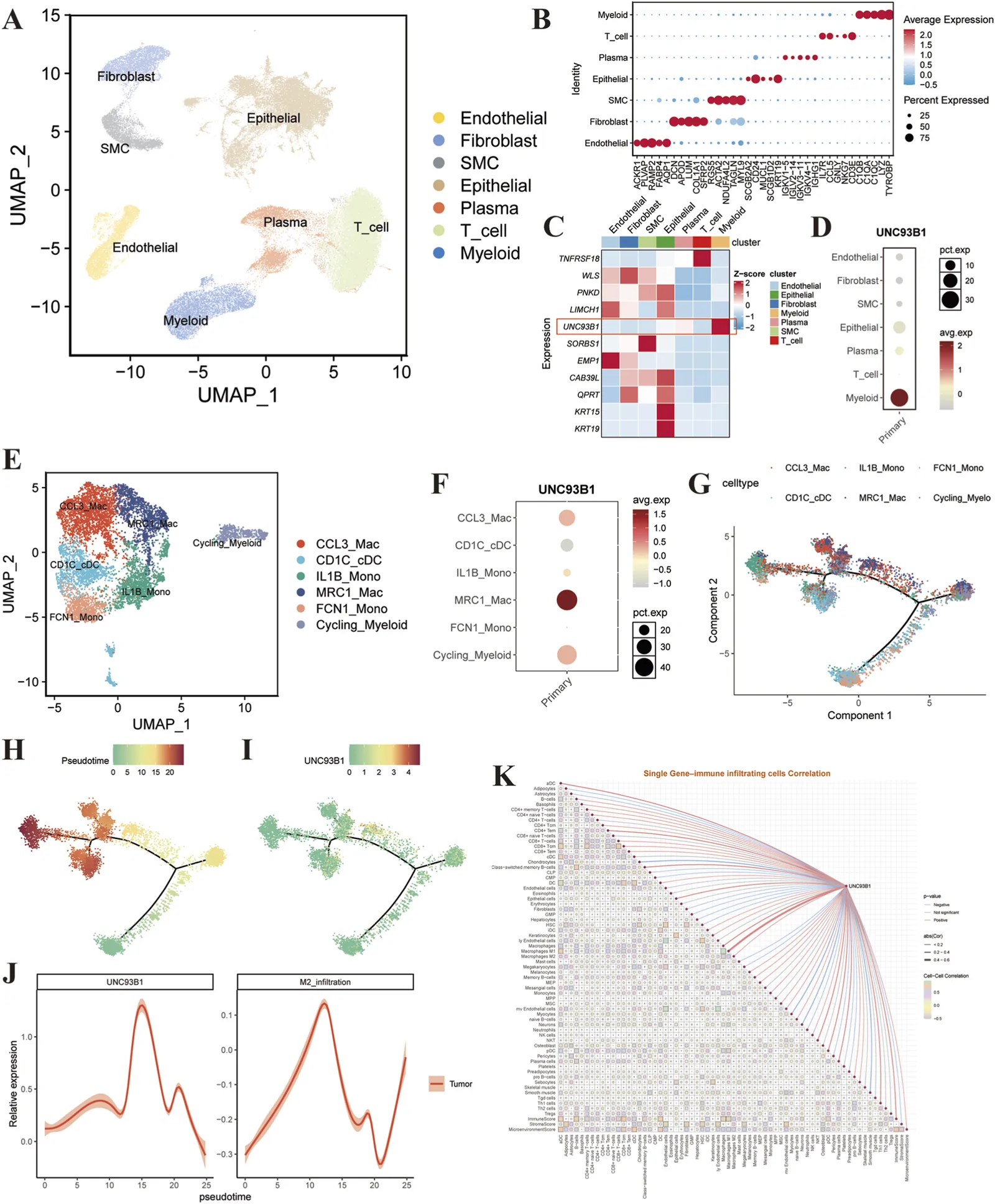
Single-cell transcriptomic analysis reveals UNC93B1-enriched myeloid and macrophage states in BRCA. **(A)** UMAP visualization of major cell populations identified in BRCA single-cell RNA-seq data, including endothelial cells, fibroblasts, smooth muscle cells, epithelial cells, plasma cells, T cells, and myeloid cells. **(B)** Dot plot showing canonical marker gene expression used for cell-type annotation. **(C)** Heatmap displaying the expression distribution of 11 LMrisk genes across major cell populations. **(D)** Dot plot illustrating cell-type-specific expression of UNC93B1, with main enrichment in myeloid cells. **(E)** UMAP visualization of re-clustered myeloid subpopulations, including CCL3_Mac, CD1C_cDC, IL1B_Mono, MRC1_Mac, FCN1_Mono, and Cycling_Myeloid. **(F)** Dot plot showing UNC93B1 expression across myeloid subclusters. **(G)** Pseudotime trajectory of myeloid subpopulations. **(H)** Distribution of pseudotime mapped onto the myeloid trajectory. **(I)** Projection of UNC93B1 expression along the myeloid pseudotime trajectory. **(J)** Dynamic trends of UNC93B1 expression and M2 macrophage infiltration score along pseudotime. **(K)** Correlation analysis between UNC93B1 expression and immune or stromal cell infiltration signatures.

To delineate the cellular context of the LMrisk model, we examined BRCA single-cell transcriptomic data and generated an integrated cellular atlas. Unsupervised clustering and UMAP visualization facilitated the identification of seven principal cell populations, namely, endothelial cells, fibroblasts, smooth muscle cells, epithelial cells, plasma cells, T cells, and myeloid cells (Figure 3A). Cell-type annotation was corroborated by the use of canonical lineage markers, including ACKR1, PLVAP, and RAMP2 for endothelial cells; DCN, APOD, LUM, and COL1A1 for fibroblasts; ACTA2, TAGLN, and MYL9 for smooth muscle cells; SCGB2A2, CD24, MUCL1, and KRT19 for epithelial cells; immunoglobulin-related genes for plasma cells; IL7R, CCL5, GNLY, NKG7, and CD3E for T cells; and C1QA, C1QB, C1QC, LYZ, and TYROBP for myeloid cells (Figure 3B).

Mapping the 11 LMrisk genes onto this cellular atlas revealed marked cell-type specificity rather than uniform expression across the tumor ecosystem (Figure 3C). Among these genes, UNC93B1 showed predominant enrichment in myeloid cells, with relatively limited expression in epithelial and stromal compartments (Figure 3D). This distribution suggests that UNC93B1 may represent an immune-contextualized component of the LMrisk model, linking lipid metabolism-related prognostic risk to the myeloid compartment of the BRCA microenvironment.

Given this myeloid enrichment, we further re-clustered the myeloid compartment and identified six subpopulations: CCL3_Mac, CD1C_cDC, IL1B_Mono, MRC1_Mac, FCN1_Mono, and Cycling_Myeloid (Figure 3E). UNC93B1 was preferentially expressed in myeloid/macrophage populations, particularly in the MRC1_Mac cluster, which is associated with an M2-like macrophage phenotype. UNC93B1-high myeloid/macrophage cells may represent an MRC1-positive TAM-related state associated with M2-like macrophage features (Figure 3F). Pseudotime analysis revealed a continuous myeloid differentiation trajectory, along which UNC93B1 displayed stage-dependent expression and showed a trend similar to the M2 infiltration score (Figures 3G–J). These findings indicate that UNC93B1-positive myeloid states may be associated with macrophage polarization and dynamic immune remodeling in BRCA.

### 3.6 UNC93B1-high myeloid states demonstrate innate immune, phagolysosomal, and immunoregulatory programs

To characterize the biological programs associated with UNC93B1, we compared UNC93B1-high and UNC93B1-low myeloid states. Differential expression analysis revealed a distinct UNC93B1-associated transcriptional profile (Supplementary Figure S4A). GO enrichment analysis showed that UNC93B1-associated genes were enriched in immune response-activating signaling, antigen processing and presentation, regulation of inflammatory response, reactive oxygen species-related processes, and cellular oxidant detoxification (Supplementary Figure S4B). Rather than indicating a canonical M1 phenotype alone, these programs likely reflect a broader innate immune and phagolysosomal activation state within tumor-associated myeloid cells.

KEGG enrichment further emphasized pathways such as lysosome, phagosome, antigen processing and presentation, IL-17 signaling, lipid and atherosclerosis, cholesterol metabolism, and ECM–receptor interaction (Supplementary Figure S4C). In conjunction with the enrichment of UNC93B1 in MRC1-positive macrophage states and its temporal alignment with the M2 infiltration score, these findings imply that UNC93B1-high cells may constitute an immunoregulatory TAM-like phenotype. This phenotype is characterized by innate immune sensing, phagolysosomal activity, oxidative stress adaptation, lipid-related inflammatory remodeling, and M2-like polarization, rather than representing a purely M1-like inflammatory population.

**FIGURE 4:**
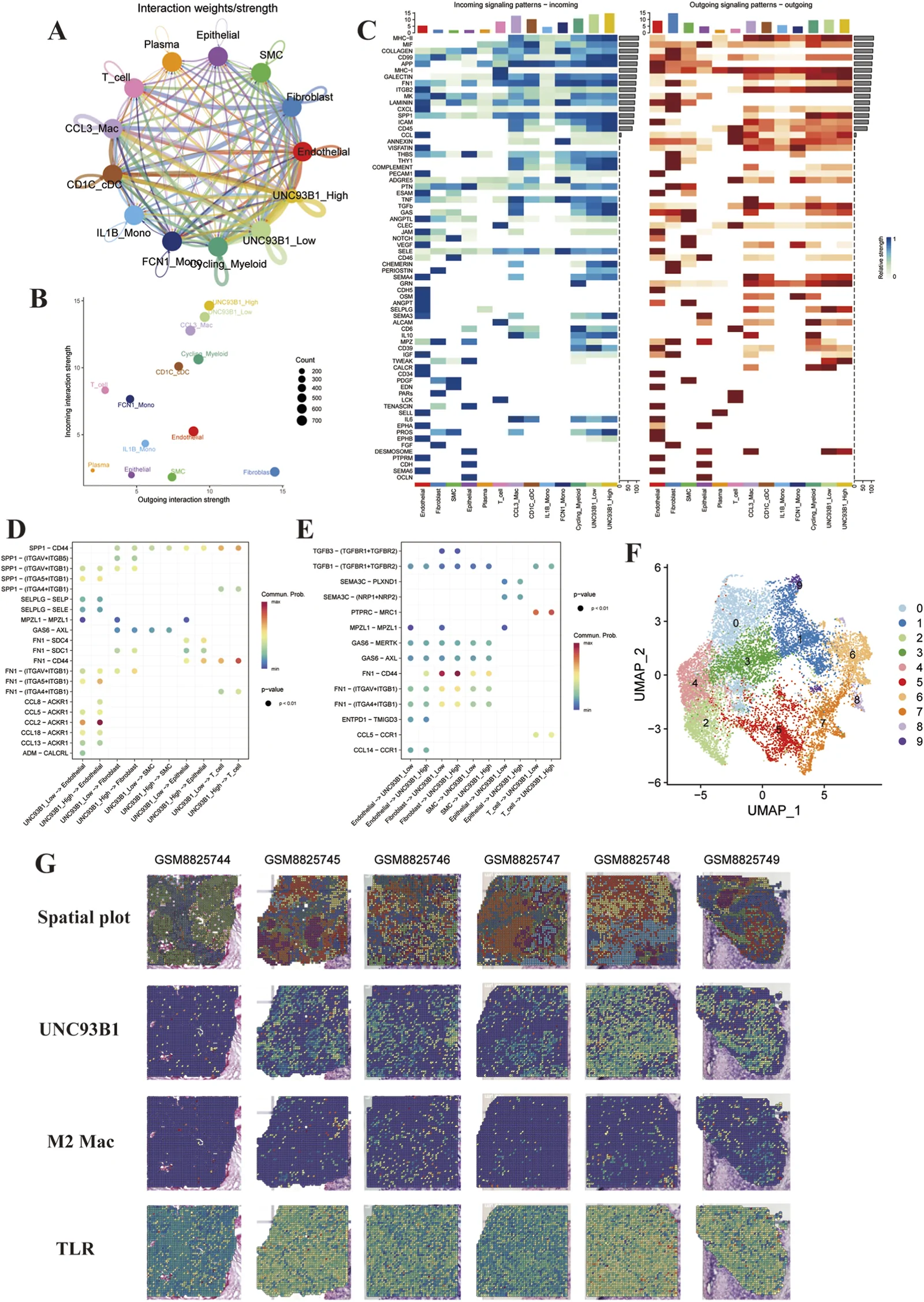
Cell–cell communication and spatial transcriptomics support a UNC93B1-centered macrophage–microenvironment axis. **(A)** Global cell–cell communication network displaying interaction weights and strengths among major cell populations and UNC93B1-defined cell states. **(B)** Scatter plot illustrating incoming and outgoing interaction strengths across cell populations, emphasizing the signaling activity of UNC93B1-high and UNC93B1-low states. **(C)** Heatmaps depicting incoming and outgoing signaling patterns across cell types and UNC93B1-defined states. **(D)** Ligand–receptor interactions from UNC93B1-defined cells to endothelial, fibroblast, smooth muscle, epithelial, and T-cell compartments. **(E)** Ligand–receptor interactions from stromal, epithelial, endothelial, and T-cell compartments toward UNC93B1-defined cells. **(F)** UMAP visualization of spatial transcriptomic clusters in BRCA tissue sections. **(G)** Spatial feature plots illustrating the distribution of UNC93B1, M2 macrophage-related signals, and Toll-like receptor-related signals across BRCA spatial transcriptomic samples.

We further examined intercellular communication between UNC93B1-defined myeloid states and other cellular compartments. Global interaction analysis demonstrated extensive communication among endothelial cells, fibroblasts, epithelial cells, T cells, macrophage/monocyte populations, and UNC93B1-high/low states (Supplementary Figure S4D; Figure 4A). Quantitative comparison of incoming and outgoing interaction strengths indicated that UNC93B1-high cells held an active signaling position within the cellular communication network (Figure 4B). Analyses of incoming and outgoing signaling patterns further revealed that UNC93B1-associated states participated in multiple immune, adhesion, extracellular matrix, and cytokine-related pathways, including MHC-I/MHC-II, MIF, COLLAGEN, FN1, SPP1, ICAM, CCL, TGFβ, IL10, and IL6 signaling programs (Figure 4C).

### 3.7 Cell–cell communication and spatial transcriptomics support an UNC93B1-centered macrophage–microenvironment axis

To further delineate the signaling pathways potentially linking UNC93B1-positive myeloid states with the adjoining tumor microenvironment, we analyzed ligand–receptor interactions involving populations characterized by high and low UNC93B1 expression. The outgoing communication from UNC93B1-defined cells to endothelial, fibroblast, smooth muscle, epithelial, and T-cell compartments encompassed several matrix- and immune-regulatory interactions, including SPP1–CD44, SPP1–integrin complexes, FN1–integrin complexes, FN1–CD44, GAS6–AXL, SELPLG–SELE/SELP, and multiple CCL–ACKR1 interactions (Figure 4D). Conversely, incoming signals from stromal, epithelial, endothelial, and T-cell compartments directed towards UNC93B1-defined cells included TGFB1/TGFB3–TGFBR1/TGFBR2, GAS6–AXL/MERTK, FN1–integrin/CD44, PTPRC–MRC1, and SEMA3C–NRP/PLXND1 interactions (Figure 4E). These ligand–receptor patterns imply that UNC93B1-positive myeloid states may engage in reciprocal crosstalk with stromal and tumor compartments via extracellular matrix remodeling, chemokine signaling, TGFβ-mediated immunoregulation, and macrophage-associated receptor pathways.

Supplementary pathway-level communication analysis further substantiated this interpretation. The comprehensive cell–cell communication network demonstrated extensive signaling exchanges among immune and stromal populations. Concurrently, pathway-specific analyses identified CCL, SPP1, FN1, and TGFβ signaling as primary communication axes linking macrophage/monocyte states with endothelial, fibroblast, epithelial, T-cell, and UNC93B1-defined populations (Supplementary Figure S4D–L). These pathways are highly consistent with processes such as macrophage recruitment, matrix remodeling, adhesion-dependent signaling, and immunosuppressive polarization, thereby suggesting that UNC93B1-associated myeloid states may influence the formation of a permissive tumor microenvironment through coordinated intercellular communication.

Finally, spatial transcriptomic analysis was conducted to validate the tissue-level localization of the UNC93B1-associated immune program. Spatial clustering identified multiple spatial domains across BRCA tissue sections (Figure 4F). Spatial feature mapping demonstrated that UNC93B1 signals partially overlapped with M2 macrophage-enriched and TLR-related spatial patterns across various samples (Figure 4G). Given the current resolution limitations of spatial transcriptomic profiling, these data should be interpreted as supportive evidence for spatial proximity rather than definitive single-cell-level co-localization. Nonetheless, this spatial pattern aligns with the single-cell findings and indicates that UNC93B1 may be associated with a spatially organized myeloid niche characterized by M2-like macrophage infiltration and innate immune/TLR-related activity. Collectively, these single-cell, cell–cell communication, and spatial transcriptomic analyses delineate a UNC93B1-centered macrophage–microenvironment axis that potentially links lipid metabolism-related prognostic risk with inflammatory signaling, M2-like polarization, extracellular matrix remodeling, and immune niche organization in BRCA.

### 3.8 Multilevel validation identifies UNC93B1 as a clinically relevant marker in BRCA

**FIGURE 5:**
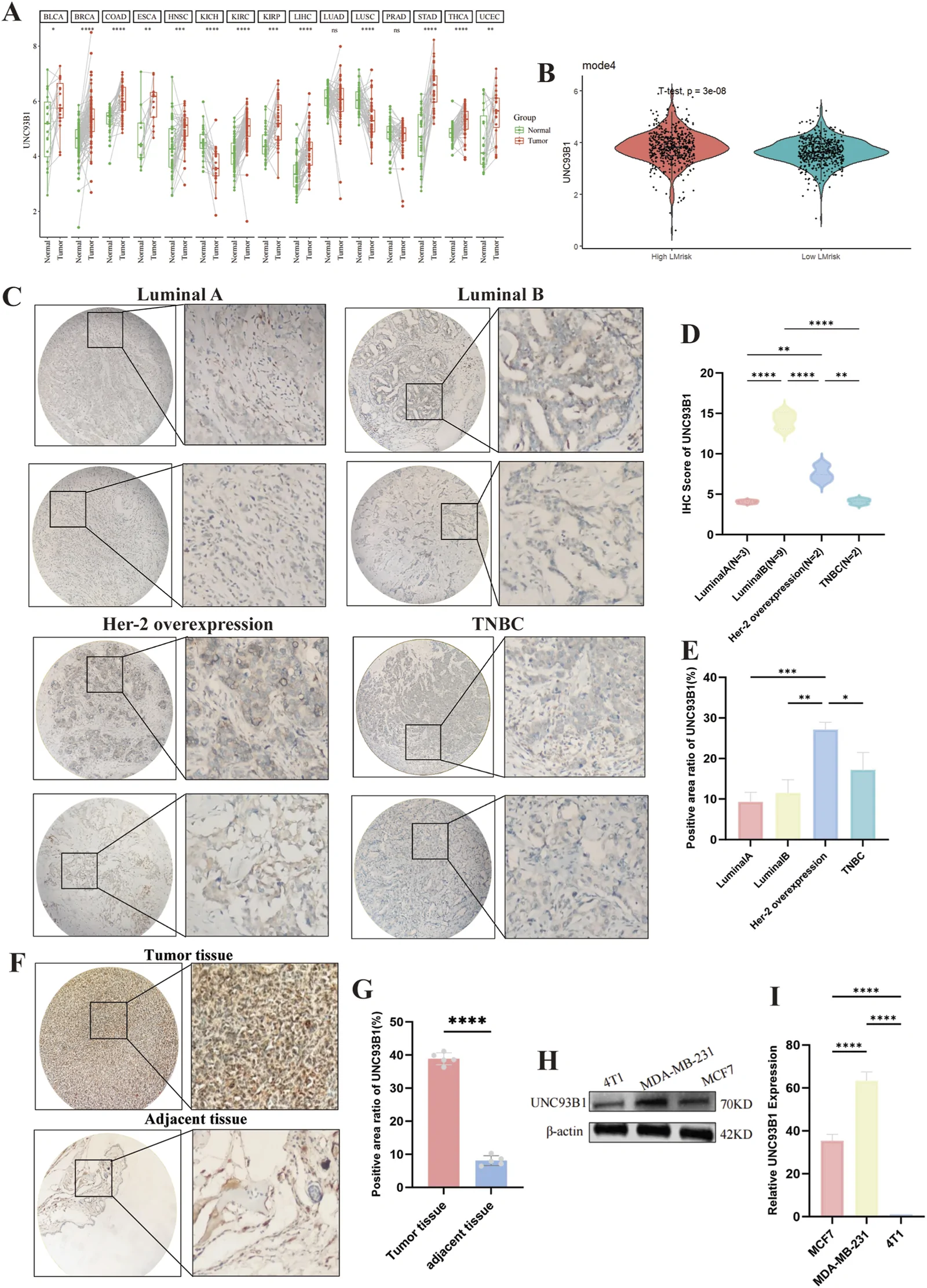
Multilevel validation identifies UNC93B1 as a clinically relevant marker in BRCA. **(A)** Pan-cancer expression analysis showing differential UNC93B1 expression between tumor and normal tissues across multiple cancer types. **(B)** Violin plot displaying higher UNC93B1 expression in the high-LMrisk group compared to the low-LMrisk group. **(C)** Representative IHC images of UNC93B1 staining across major BRCA molecular subtypes, including Luminal A, Luminal B, HER2-overexpression, and TNBC. **(D)** Quantification of UNC93B1 IHC H-scores across BRCA molecular subtypes. **(E)** Quantification of the UNC93B1-positive staining area across BRCA molecular subtypes. **(F)** Representative IHC images of UNC93B1 staining in MCF-7 xenograft tumor tissues and adjacent non-tumor tissues from nude mice. **(G)** Quantification of UNC93B1-positive staining area in xenograft tumor and adjacent non-tumor tissues. **(H)** Western blot analysis of UNC93B1 protein expression in breast cancer cell lines. **(I)** qRT-PCR analysis of UNC93B1 mRNA expression in breast cancer cell lines. Statistical significance is indicated in the panels.

To further evaluate the clinical relevance of UNC93B1 in breast cancer, we undertook comprehensive validation procedures, including pan-cancer expression profiling, LMrisk-based stratification, subtype-specific immunohistochemical analysis, and cellular experiments. The pan-cancer analysis demonstrated that UNC93B1 exhibited differential expression between tumor and normal tissues across various cancer types, suggesting that its dysregulation may be a common event linked to tumorigenesis (Figure 5A). In breast cancer, patients classified within the high-LMrisk group showed significantly elevated UNC93B1 expression compared to those in the low-LMrisk group, thereby establishing an association between UNC93B1 and a lipid metabolism-related high-risk phenotype (Figure 5B).

IHC analysis further demonstrated notable heterogeneity in UNC93B1 protein expression, which varies depending on the subtype. The strongest staining was observed in the HER2-overexpression subtype, followed by triple-negative breast cancer (TNBC). Conversely, Luminal A and Luminal B tumors exhibited relatively lower levels of expression (Figures 5C–E; Supplementary Figure S5A–H). Additionally, in nude mouse xenografts derived from MCF-7 cells, IHC analysis revealed more intense UNC93B1 staining in the tumor tissues compared to the adjacent non-tumorous tissues surrounding the tumor mass (Figures 5F,G).

Consistently, Western blotting and qRT-PCR analyses showed that UNC93B1 expression was higher in the highly invasive MDA-MB-231 cells than in MCF-7 and 4T1 cells (Figures 5H,I). Collectively, these findings demonstrate that UNC93B1 is upregulated in BRCA, enriched in aggressive molecular subtypes, associated with high LMrisk status, and preferentially expressed in invasive breast cancer cells, supporting its potential role as a clinically relevant lipid metabolism-associated immune-regulatory marker.

### 3.9 UNC93B1 promotes proliferative, migratory, and invasive phenotypes in BRCA cells

**FIGURE 6:**
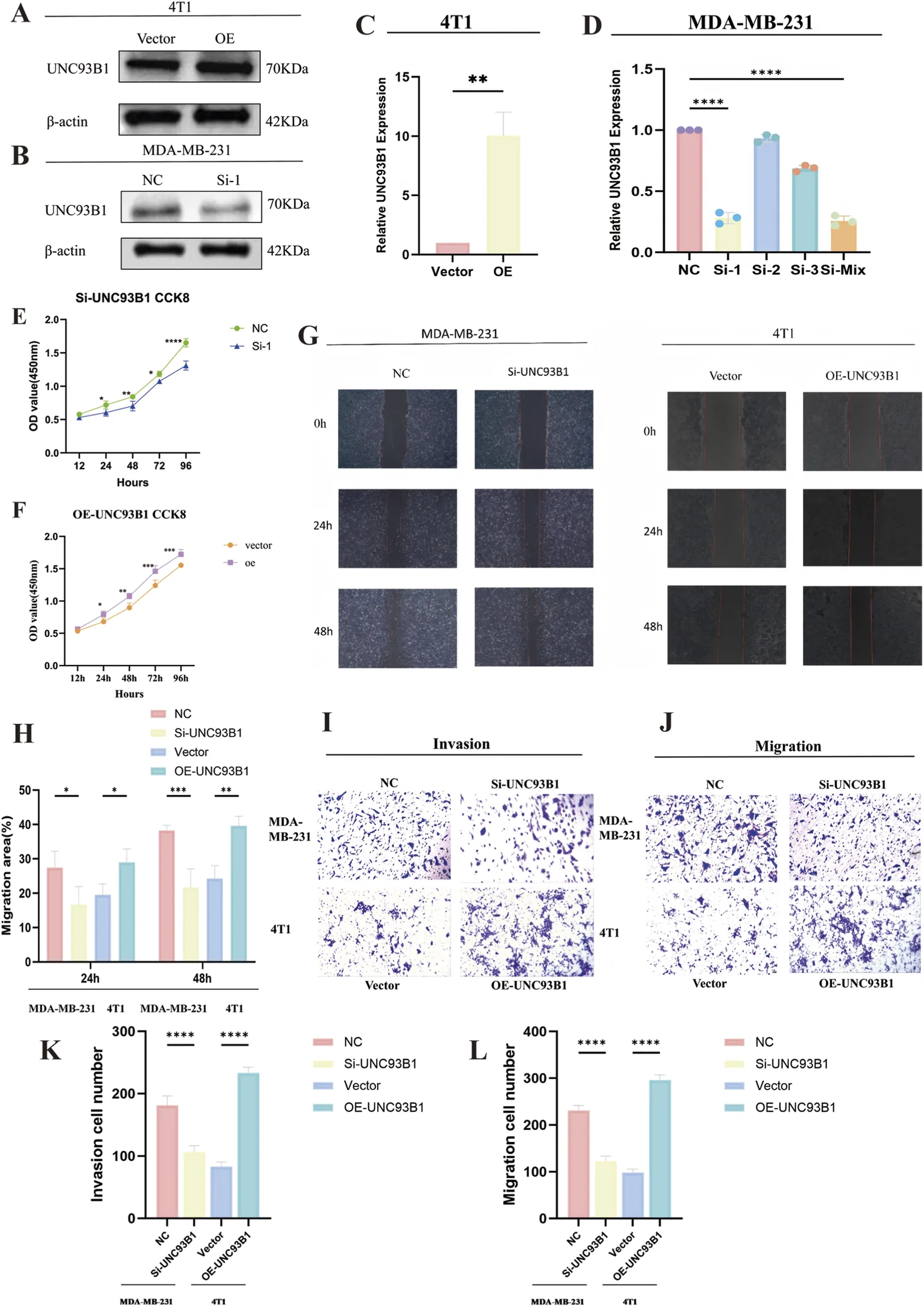
UNC93B1 promotes proliferation, migration, and invasion of breast cancer cells. **(A)** Western blot analysis of UNC93B1 protein expression in 4T1 cells transfected with UNC93B1 overexpression plasmid or empty vector. **(B)** Western blot analysis of UNC93B1 protein expression in MDA-MB-231 cells transfected with UNC93B1 siRNA or negative control siRNA. **(C)** qRT-PCR analysis of UNC93B1 mRNA expression in UNC93B1-overexpressing 4T1 cells. **(D)** qRT-PCR analysis of UNC93B1 mRNA expression in UNC93B1-knockdown MDA-MB-231 cells. **(E)** CCK-8 assay showing the effect of UNC93B1 knockdown on MDA-MB-231 cell proliferation. **(F)** CCK-8 assay showing the effect of UNC93B1 overexpression on 4T1 cell proliferation. **(G,H)** Scratch wound-healing assays and quantitative analysis showing the migratory ability of UNC93B1-knockdown MDA-MB-231 cells and UNC93B1-overexpressing 4T1 cells. **(I,K)** Transwell invasion assays and quantitative analysis of UNC93B1-knockdown MDA-MB-231 cells and UNC93B1-overexpressing 4T1 cells. **(J,L)** Transwell migration assays and quantitative analysis of UNC93B1-knockdown MDA-MB-231 cells and UNC93B1-overexpressing 4T1 cells. Data are presented as mean ± SD where applicable; statistical significance is indicated in the panels.

To elucidate the functional role of UNC93B1 in BRCA progression, we established two complementary genetic manipulation models in breast cancer cells: siRNA-mediated UNC93B1 knockdown in human MDA-MB-231 cells and UNC93B1 overexpression in mouse 4T1 cells. Western blot analysis confirmed effective UNC93B1 overexpression in 4T1 cells and successful UNC93B1 knockdown in MDA-MB-231 cells at the protein level, with consistent β-actin expression within each panel indicating equivalent protein loading (Figures 6A,B). Quantitative real-time PCR further corroborated the substantial upregulation of UNC93B1 in the overexpression cohort and its significant downregulation following siRNA-mediated silencing (Figures 6C,D). Among the siRNA sequences tested, si-1 demonstrated the most potent knockdown efficiency and was consequently selected for subsequent functional assays.

Functionally, UNC93B1 depletion in MDA-MB-231 cells significantly impaired malignant phenotypes, whereas UNC93B1 overexpression in 4T1 cells exerted the opposite effects. CCK-8 assays demonstrated that UNC93B1 knockdown suppressed cellular proliferation, while UNC93B1 overexpression enhanced proliferative capacity over time (Figures 6E,F). Consistently, scratch assays showed reduced migratory closure following UNC93B1 silencing and accelerated migration after UNC93B1 overexpression (Figures 6G,H). Transwell assays further confirmed that UNC93B1 knockdown decreased both migratory and invasive cell quantities, whereas UNC93B1 overexpression increased these capacities (Figures 6I–L). Collectively, these loss- and gain-of-function experiments demonstrate that UNC93B1 actively promotes BRCA cell proliferation, migration, and invasion, thereby supporting its role as a functional driver of malignant progression.

### 3.10 UNC93B1 drives BRCA cell–macrophage crosstalk through an IL-6/STAT3-dependent paracrine axis

**FIGURE 7:**
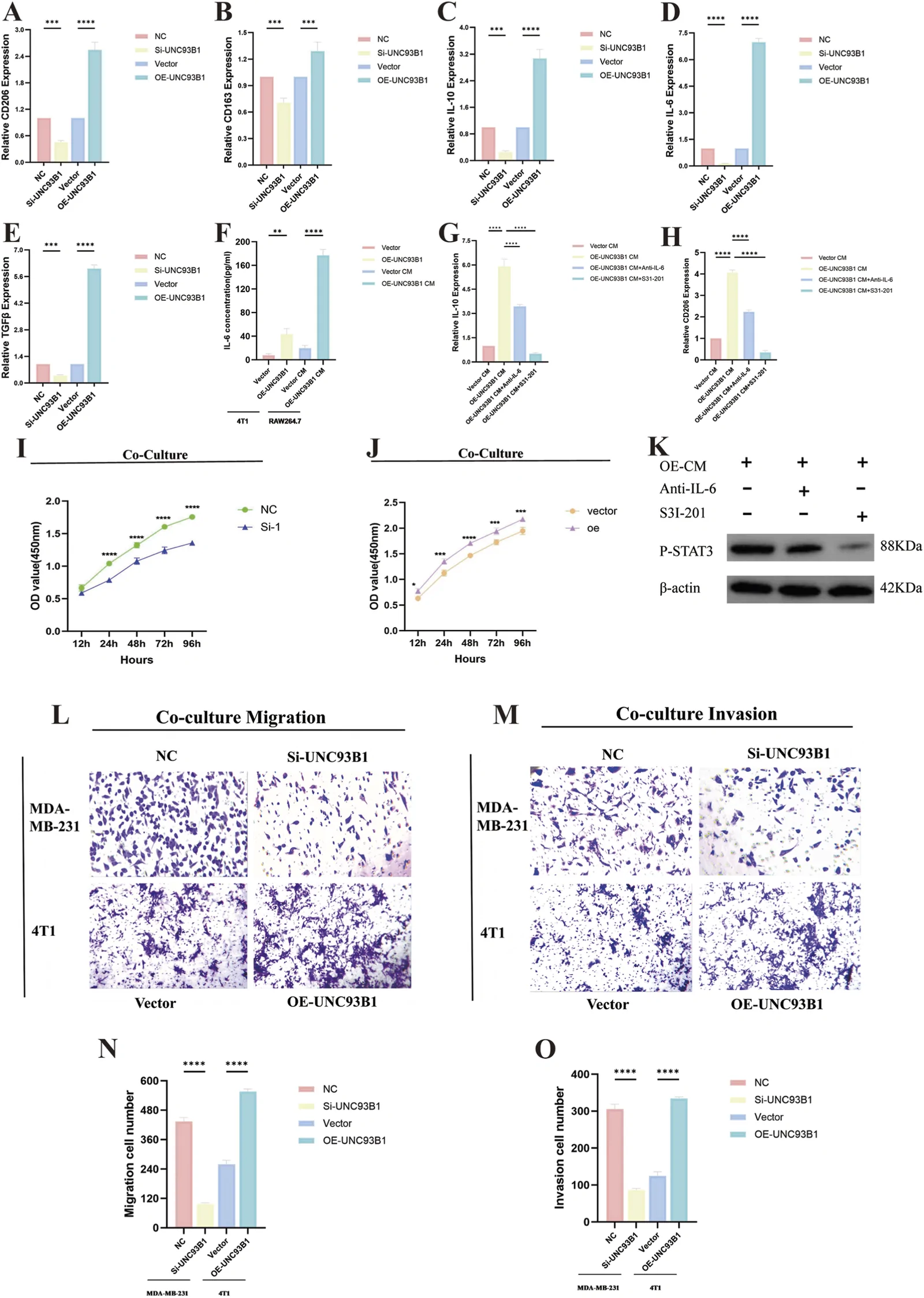
UNC93B1 promotes tumor cell–macrophage crosstalk and M2-like macrophage polarization through IL-6/STAT3 signaling. **(A–E)** qRT-PCR analysis of M2-like macrophage markers in macrophages after tumor cell–macrophage co-culture. THP-1-derived macrophages were co-cultured with MDA-MB-231 cells transfected with negative control siRNA or si-UNC93B1. RAW264.7 macrophages were co-cultured with 4T1 cells transfected with empty vector or UNC93B1 overexpression plasmid. The expression levels of CD206, CD163, IL-10, IL-6, and TGF-β were measured to evaluate M2-like macrophage polarization. **(F)** IL-6 concentrations in conditioned media collected from Vector- or OE-UNC93B1-transfected 4T1 cells and in culture supernatants from RAW264.7 macrophages treated with the corresponding conditioned media, as determined by ELISA. **(G,H)** qRT-PCR analysis showing the effects of IL-6 neutralization and STAT3 inhibition on M2-like macrophage marker expression in RAW264.7 macrophages treated with conditioned medium from UNC93B1-overexpressing 4T1 cells. **(I,J)** CCK-8 assays demonstrating the proliferative effects of UNC93B1-mediated tumor cell–macrophage crosstalk in MDA-MB-231/THP-1-derived macrophage and 4T1/RAW264.7 co-culture models. **(K)** Western blot analysis of STAT3 phosphorylation in RAW264.7 macrophages treated with conditioned medium from vector-control or UNC93B1-overexpressing 4T1 cells, with or without IL-6 neutralization or STAT3 inhibition. **(L–O)** Transwell migration and invasion assays illustrating the functional effects of UNC93B1-mediated tumor cell–macrophage crosstalk. MDA-MB-231 cells were used for UNC93B1 knockdown experiments with THP-1-derived macrophages, while 4T1 cells were used for UNC93B1 overexpression experiments with RAW264.7 macrophages. Data are shown as the mean ± SD from at least three independent experiments. *P < 0.05, **P < 0.01, ***P < 0.001, and ****P < 0.0001.

To ascertain the immunoregulatory function of UNC93B1 within the BRCA microenvironment, we developed tumor cell–macrophage co-culture systems and assessed macrophage polarization, IL-6 secretion, STAT3 activation, and malignant phenotypes of tumor cells. In the MDA-MB-231/THP-1-derived macrophage co-culture model, UNC93B1 knockdown in MDA-MB-231 cells significantly reduced the expression of M2-associated markers in THP-1-derived macrophages, including CD206, CD163, IL-10, IL-6, and TGF-β, compared with the control group. In contrast, in the 4T1/RAW264.7 co-culture model, UNC93B1 overexpression in 4T1 cells markedly increased the expression of these M2-related markers in RAW264.7 macrophages (Figures 7A–E).

Consistent with this polarization phenotype, ELISA analysis showed that conditioned medium collected from UNC93B1-overexpressing 4T1 cells contained significantly higher IL-6 levels than conditioned medium from vector-control 4T1 cells, indicating that UNC93B1 promotes tumor-derived IL-6 secretion (Figure 7F). To further investigate whether the IL-6/STAT3 pathway mediates this macrophage response, conditioned medium from UNC93B1-overexpressing cells was combined with either an IL-6-neutralizing antibody or the STAT3 inhibitor S3I-201. Both treatments significantly reduced the induction of CD206 and IL-10, and Western blotting confirmed that conditioned medium-induced STAT3 phosphorylation was suppressed by IL-6 neutralization or STAT3 inhibition (Figures 7G,H,K). These findings suggest that UNC93B1-dependent M2-like macrophage polarization is at least partly driven by tumor-derived IL-6 and downstream STAT3 activation.

Functionally, UNC93B1 also augmented the malignant behavior of BRCA cells under co-culture conditions. CCK-8 assays indicated that knockdown of UNC93B1 diminished tumor cell proliferation, whereas overexpression of UNC93B1 enhanced proliferative capacity (Figures 7I,J). Transwell assays further demonstrated that UNC93B1 knockdown reduced tumor cell migration and invasion, while UNC93B1 overexpression markedly increased both phenotypes (Figures 7L–O). Collectively, these findings support a model wherein tumor cell-derived UNC93B1 promotes IL-6 secretion, activates macrophage STAT3 signaling, induces M2-like macrophage polarization, and reinforces a paracrine tumor–macrophage circuit that facilitates BRCA cell proliferation, migration, and invasion.

### 3.11 UNC93B1 promotes EMT via an IL-6/STAT3-dependent tumor–macrophage feedback loop

**FIGURE 8:**
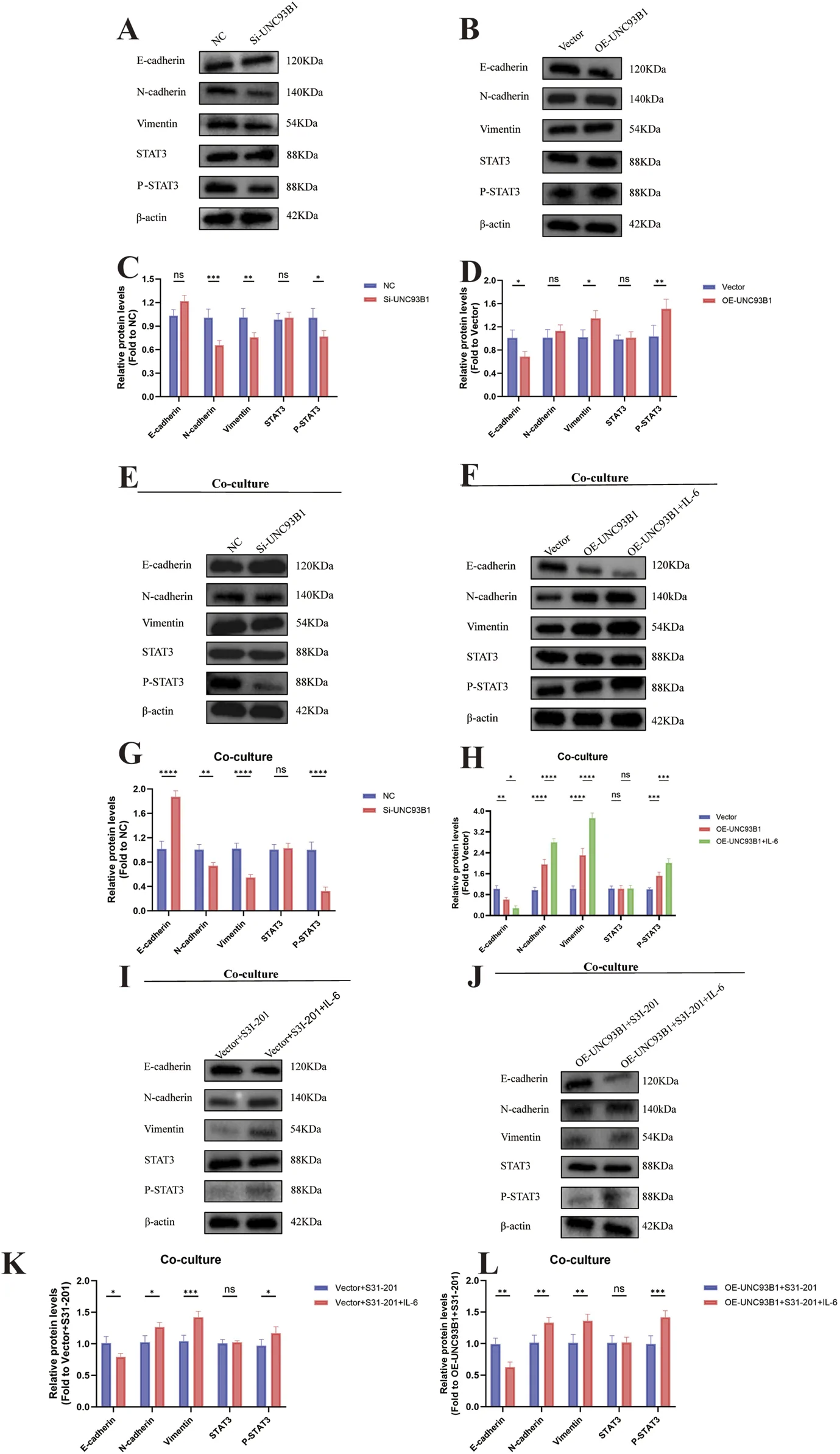
UNC93B1 promotes EMT through IL-6/STAT3-dependent tumor cell–macrophage communication. **(A–D)** Western blot analysis of EMT-related proteins and STAT3 activation in monocultured breast cancer cells. UNC93B1 knockdown was performed in MDA-MB-231 cells, and UNC93B1 overexpression was performed in 4T1 cells. **(E–H)** Western blot analysis of EMT-related proteins and STAT3 activation in tumor cells after co-culture with macrophages. MDA-MB-231 cells, with or without UNC93B1 knockdown, were co-cultured with THP-1-derived macrophages, while 4T1 cells, with or without UNC93B1 overexpression, were co-cultured with RAW264.7 macrophages. **(I–L)** Western blot analysis showing the effects of STAT3 inhibition and IL-6 treatment in the 4T1/RAW264.7 macrophage co-culture model. Vector-control or UNC93B1-overexpressing 4T1 cells were co-cultured with RAW264.7 macrophages under treatment with S3I-201 alone or S3I-201 combined with recombinant IL-6. E-cadherin, N-cadherin, Vimentin, total STAT3, phosphorylated STAT3, and β-actin were examined. Quantification shows relative protein expression normalized to β-actin or total STAT3, as appropriate. Data are presented as the mean ± SD from at least three independent experiments. *P < 0.05, **P < 0.01, ***P < 0.001, and ****P < 0.0001.

To further elucidate the molecular mechanism by which UNC93B1 facilitates BRCA progression, we examined whether UNC93B1 modulates EMT via an IL-6/STAT3-dependent signaling pathway and enhances tumor–macrophage crosstalk within the tumor microenvironment. Under monoculture conditions, UNC93B1 knockdown in MDA-MB-231 cells resulted in increased expression of the epithelial marker E-cadherin and decreased levels of the mesenchymal markers N-cadherin and Vimentin, accompanied by reduced STAT3 phosphorylation. Conversely, UNC93B1 overexpression in 4T1 cells induced the opposite EMT-related changes, characterized by decreased E-cadherin, increased N-cadherin and Vimentin, and augmented activation of STAT3 (Figures 8A–D). Importantly, although these EMT- and p-STAT3-associated changes were observable under monoculture conditions, their overall magnitude was relatively modest, indicating that the pro-malignant role of UNC93B1 may not rely solely on tumor cell-intrinsic signaling but could be further amplified through cell–cell interactions within the tumor microenvironment.

Based on this observation, the subsequent evaluation focused on the influence of UNC93B1 on EMT regulation within a tumor cell–macrophage co-culture system. Compared with monoculture conditions, tumor cell–macrophage co-culture amplified UNC93B1-associated EMT alterations. Specifically, MDA-MB-231 cells were used for UNC93B1 knockdown experiments with THP-1-derived macrophages, whereas 4T1 cells were used for UNC93B1 overexpression experiments with RAW264.7 macrophages. In the presence of macrophages, knockdown of UNC93B1 further inhibited the expression of N-cadherin, Vimentin, and p-STAT3, while restoring E-cadherin levels. Conversely, overexpression of UNC93B1 induced a more evident EMT-promoting phenotype and further increased STAT3 phosphorylation (Figures 8E–H). These findings suggest that UNC93B1-mediated EMT activation is highly dependent on the tumor microenvironment and that its pro-tumorigenic effects are markedly intensified within the context of tumor cell–macrophage interactions.

Considering our prior discovery that UNC93B1 facilitates tumor-derived IL-6 secretion and promotes M2-like macrophage polarization, we further investigated whether IL-6 functions as a crucial paracrine mediator in the crosstalk between tumors and macrophages. In the co-culture system, the addition of exogenous IL-6 markedly amplified the EMT induced by UNC93B1 overexpression, as evidenced by reduced E-cadherin, increased N-cadherin and Vimentin levels, and heightened p-STAT3 levels (Figures 8F,H). These results substantiate the significant signaling role of IL-6 in UNC93B1-mediated tumor–macrophage communication, likely occurring via activation of STAT3-dependent EMT pathways.

To determine whether STAT3 is an essential downstream node in this signaling axis, we performed pharmacological inhibition using the STAT3 inhibitor S3I-201, combined with exogenous IL-6 rescue experiments. S3I-201 markedly reversed the EMT phenotype induced by UNC93B1 overexpression and the co-culture microenvironment, as evidenced by restored E-cadherin expression, reduced N-cadherin and Vimentin levels, and strong suppression of p-STAT3 (Figures 8I–L). Importantly, under STAT3-inhibited conditions, supplementation with exogenous IL-6 failed to fully restore EMT marker changes or STAT3 phosphorylation. This indicates that IL-6-mediated EMT amplification requires functional STAT3 signaling and cannot bypass STAT3 to directly drive EMT.

Collectively, these results support a microenvironment-amplified mechanism in which upregulated UNC93B1 in tumor cells promotes IL-6-related paracrine signaling, strengthens macrophage-involved tumor microenvironmental crosstalk, and further amplifies EMT programs through STAT3 activation. This UNC93B1–IL-6–STAT3 feedback axis links tumor cell-intrinsic signaling with macrophage-mediated microenvironmental remodeling, thereby promoting EMT, migration, invasion, and malignant progression in BRCA.

## 4 Discussion

The progression of BRCA is increasingly recognized as a consequence of coordinated metabolic reprogramming and remodeling of the tumor immune microenvironment, rather than solely due to isolated oncogenic alterations (Hanahan, 2022; Wan et al., 2025). In this framework, lipid metabolism has transcended its traditional function as a provider of bioenergetic and biosynthetic resources to serve as a signaling hub that governs tumor plasticity, immune evasion, therapeutic resistance, and metastatic potential (Koundouros and Poulogiannis, 2020; Luo et al., 2017; Wang et al., 2024). Among these mechanisms, EMT constitutes a pivotal cellular program through which tumor cells acquire migratory and invasive phenotypes, while the remodeling of TAMs fosters an inflammatory and immunosuppressive niche essential for this transition (Grasset et al., 2022; Munir et al., 2021; Thiery et al., 2009). Importantly, these two processes are metabolically interconnected: M2-like macrophages predominantly depend on fatty acid oxidation and oxidative phosphorylation, whereas lipid cues derived from tumors can further promote M2-biased polarization (Chen et al., 2021; Qiao et al., 2023; Zhang et al., 2024). Consequently, lipid metabolic reprogramming, macrophage polarization, and EMT should not be regarded as isolated features of BRCA progression, but rather as synergistic components within a malignant ecosystem that facilitates invasion, metastasis, and immune evasion.

IL-6/STAT3 signaling constitutes a central inflammatory axis through which the metabolic–immune ecosystem may be translated into invasive tumor behavior. IL-6, derived from both tumor cells and infiltrating macrophages, activates the IL-6R/gp130–JAK–STAT3 pathway, leading to sustained STAT3 phosphorylation, transcriptional activation of EMT-related programs, macrophage immunoregulatory polarization, and attenuation of antitumor immune surveillance (Heo et al., 2016; Hu Z. et al., 2024; Manore et al., 2022). This bidirectional cytokine signaling establishes a feed-forward circuit in which inflammatory remodeling and EMT plasticity mutually reinforce each other. Nevertheless, despite the recognized importance of lipid metabolism, TAM polarization, and IL-6/STAT3 signaling in BRCA progression, the upstream molecular regulators linking lipid metabolism-associated risk states to macrophage-driven immunosuppression and EMT activation remain inadequately characterized (Arner and Rathmell, 2023; Mehta et al., 2021; Pascual and Benitah, 2024). The identification of such regulatory nodes is crucial for comprehending how metabolic risk factors are converted into a spatially organized, immune-suppressive, and pro-metastatic tumor microenvironment.

Although previous studies have independently shown that IL-6/STAT3 signaling contributes to EMT, macrophage polarization, and tumor progression, most have focused either on tumor cell-intrinsic inflammatory signaling or on macrophage-derived cytokine effects in isolation (Fu et al., 2017; Sullivan et al., 2009; Zhao et al., 2023). In contrast, the present study links lipid metabolism-related risk stratification with single-cell-defined macrophage states, spatial immune organization, and functional tumor–macrophage communication. This integrated approach allowed us to position UNC93B1 within a cross-cellular regulatory circuit, where tumor-cell-derived IL-6 activates macrophage STAT3 signaling and promotes M2-like polarization, while macrophage-derived IL-6 reciprocally reinforces STAT3-dependent EMT programs in tumor cells. Therefore, rather than functioning as a standalone biomarker, UNC93B1 may serve as a molecular bridge connecting metabolic risk, immune remodeling, and invasive plasticity in BRCA. From a translational perspective, the UNC93B1–IL-6–STAT3 signaling axis presents a promising therapeutic target. Although strategies targeting IL-6 or STAT3 have shown promise in various tumor models, identifying suitable patient populations remains challenging (Johnson et al., 2018; Manore et al., 2022; Sadrkhanloo et al., 2022). The lipid metabolism risk model developed in this study indicates that breast tumors with high UNC93B1 expression define a molecular subtype characterized by immune-dependent EMT plasticity. Consequently, combination therapies that target this positive feedback loop could provide novel options to overcome microenvironment-driven therapeutic resistance.

The cellular context of UNC93B1 expression requires careful interpretation. Single-cell analysis showed that UNC93B1 was predominantly enriched in myeloid/macrophage compartments, particularly in MRC1-positive macrophage states. However, these findings should not be interpreted as evidence that UNC93B1 defines a distinct macrophage subtype. Rather, UNC93B1-high myeloid/macrophage cells may reflect an activated TAM-like immunoregulatory state characterized by innate immune sensing, phagolysosomal activity, TLR-related signaling, and M2-like macrophage features. In parallel, gain- and loss-of-function experiments in breast cancer cells showed that tumor-cell UNC93B1 regulated IL-6 secretion and macrophage polarization through paracrine signaling. Thus, UNC93B1 appears to operate in a context-dependent manner: at the tumor ecosystem level, it marks a myeloid-enriched immunoregulatory niche, whereas at the functional level, tumor-cell UNC93B1 promotes IL-6/STAT3-dependent macrophage reprogramming and EMT-associated malignant behavior. The macrophage-intrinsic role of UNC93B1 remains to be directly investigated.

Several limitations must be acknowledged. Firstly, although the *in vitro* tumor cell–macrophage co-culture model effectively captures essential aspects of UNC93B1-associated paracrine communication, the tumor- and microenvironment-dependent effects of UNC93B1 inhibition require further validation within orthotopic and immune-competent breast cancer models. Secondly, although UNC93B1 was identified through a lipid metabolism-related risk framework and exhibited associations with lipid metabolism-related transcriptional programs, the current study did not directly demonstrate alterations in lipid composition, fatty acid oxidation, lipid droplet accumulation, or lipid metabolic flux following UNC93B1 perturbation in breast cancer cells or macrophages. Consequently, UNC93B1 should presently be regarded as a lipid metabolism-associated immune regulatory node rather than a confirmed direct regulator of lipid metabolism. Future investigations employing lipidomic profiling, fatty acid oxidation assays, lipid droplet staining, metabolic flux analysis, and orthotopic or immune-competent breast cancer models are necessary to ascertain whether UNC93B1 directly contributes to lipid metabolic remodeling in tumor cells and macrophages. Thirdly, the spatial transcriptomic data support the spatial proximity between UNC93B1 expression, M2 macrophage-enriched regions, and TLR-related activity; however, higher-resolution spatial profiling or multiplex immunofluorescence will be required to confirm cell-type-specific co-localization. Lastly, large subtype-stratified clinical cohorts are required to determine the relationship among UNC93B1 expression, molecular subtype, macrophage infiltration, STAT3 activation, therapeutic response, and patient prognosis. In particular, whether UNC93B1 functions as a prognostic biomarker across all breast cancer subtypes or mainly in HER2-enriched and triple-negative breast cancer remains to be clarified. Future studies using multicenter cohorts, prospective samples, and predefined modeling pipelines will be required to confirm the generalizability and clinical utility of the diagnostic nomogram.

Finally, the upstream molecular mechanism by which UNC93B1 regulates IL-6 production remains incompletely defined. Although our data show that UNC93B1 knockdown decreases IL-6 secretion and attenuates STAT3-mediated macrophage M2-like polarization, we did not directly determine whether this effect is mediated through TLR-related signaling, NF-κB/MAPK activation, IL-6 promoter activity, or other transcriptional regulatory mechanisms. Additionally, macrophage-intrinsic UNC93B1 function was inferred from single-cell transcriptomic enrichment but was not directly tested by macrophage-specific UNC93B1 knockdown or knockout. Future studies using macrophage-specific UNC93B1 perturbation, IL6 promoter reporter assays, and *in vivo* models are necessary to define the precise role of UNC93B1 in macrophage states and IL-6 production.

In summary, this study identifies UNC93B1 as an immune regulatory node associated with lipid metabolism-related risk, macrophage-enriched immune remodeling, and EMT activation in breast cancer. By integrating transcriptomic modeling, single-cell and spatial analyses, and functional validation, we propose a tumor–macrophage feedback model in which tumor-cell UNC93B1 enhances IL-6/STAT3-dependent paracrine signaling, thereby promoting M2-like macrophage polarization and EMT-associated malignant progression. These findings provide a mechanistic framework for understanding how a lipid metabolism-associated risk state may be linked to immune remodeling and invasive tumor phenotypes through an inflammatory tumor–macrophage circuit. Further validation in larger clinically annotated cohorts, lipidomic and metabolic functional assays, and orthotopic or immune-competent *in vivo* breast cancer models will be required to clarify the prognostic, metabolic, and therapeutic relevance of the UNC93B1–IL-6/STAT3 axis in aggressive breast cancer.

## Data Availability

The original contributions presented in the study are included in the article/Supplementary Material, further inquiries can be directed to the corresponding author.
