# Supplementary material for: UNC93B1 drives IL-6/STAT3-dependent tumor–macrophage crosstalk and epithelial–mesenchymal transition in breast cancer
Source: Front Pharmacol. 2026 Aug 6;17:1887125. doi: 10.3389/fphar.2026.1887125 (PMC13490162; doi:10.3389/fphar.2026.1887125)
Supplement: Supplementary file 1 [file DataSheet1.docx]

**Supplementary Materials**

1. **Supplementary Figures and Figure Legends**

Supplementary Figure S1. Quality assessment, network topology, and machine-learning feature prioritization for lipid metabolism-related gene screening.

(A) Distribution of adjusted P values from differential expression analysis in the TCGA-BRCA cohort.(B) Dispersion estimates showing the mean–dispersion relationship of transcriptomic data.(C) Topological overlap matrix heatmap showing the network structure and module separation among selected genes.(D) Gene interaction network visualization of selected lipid metabolism-related candidates.(E) Mean variable importance across machine-learning algorithms based on permutation analysis.(F) Reverse cumulative distribution of residuals comparing the performance stability of different machine-learning models.

Supplementary Figure S2. Construction and validation of the LMrisk prognostic model.

(A) Univariate Cox regression analysis identifying lipid metabolism-related genes significantly associated with overall survival.(B) Cross-validation curve for LASSO Cox regression used to select the optimal penalty parameter.(C) LASSO coefficient profiles showing the shrinkage process of candidate prognostic genes.(D) Heatmap showing the expression patterns of the 11 LMrisk genes between high- and low-risk groups.(E) Survival status distribution of patients ordered by increasing LMrisk score in the TCGA-BRCA cohort.(F) LMrisk score distribution showing separation between high- and low-risk patients.(G) Survival status distribution of patients in the validation cohort ordered by increasing LMrisk score.(H) LMrisk score distribution in the validation cohort.(I) Kaplan–Meier survival analysis comparing high- and low-LMrisk groups in the validation cohort.(J) Time-dependent ROC curves evaluating the predictive performance of the LMrisk model at 1, 2, and 3 years in the validation cohort.

Supplementary Figure S3. Reinforced validation and interpretability analysis of the LMrisk model.

(A) Calibration curve of the nomogram, showing agreement between predicted and observed survival probability.(B) ROC curves comparing the predictive performance of the integrated nomogram with individual LMrisk genes.(C) ROC curve of the XGBoost-based model in the training set.(D) ROC curve of the XGBoost-based model in the validation set.(E) SHAP-based feature importance ranking showing the relative contribution of risk score and individual LMrisk genes.(F) SHAP summary plot showing the direction and magnitude of each feature’s contribution to model prediction.

Supplementary Figure S4. Functional annotation and cell–cell communication analysis of UNC93B1-associated myeloid states.

(A) Volcano plot showing differentially expressed genes between UNC93B1-high and UNC93B1-low cell states.(B) GO biological process enrichment analysis of UNC93B1-associated differentially expressed genes.(C) KEGG pathway enrichment analysis showing immune-, lysosome-, phagosome-, lipid-, and extracellular matrix-related pathways associated with UNC93B1-high states.(D) Global cell–cell communication network showing the number of interactions among major cell populations and UNC93B1-defined cell states.(E) Circular plot showing the CCL signaling network among tumor, stromal, immune, and UNC93B1-defined cell states.(F) Circular plot showing the SPP1 signaling network.(G) Circular plot showing the FN1 signaling network.(H) Circular plot showing the TGFβ signaling network.(I) Bubble plot showing source–target ligand–receptor interactions within the CCL signaling pathway.(J) Bubble plot showing source–target ligand–receptor interactions within the SPP1 signaling pathway.(K) Bubble plot showing source–target ligand–receptor interactions within the FN1 signaling pathway.(L) Bubble plot showing source–target ligand–receptor interactions within the TGFβ signaling pathway.

Supplementary Figure S5. Representative immunohistochemical images of UNC93B1 expression in BRCA molecular subtypes.

(A) Representative UNC93B1 IHC staining images of Luminal A breast cancer tissues. (B-E) Representative UNC93B1 IHC staining images of Luminal B breast cancer tissues. (F-H) Representative UNC93B1 IHC staining images of HER2-overexpression breast cancer tissues.

2. **Supplementary Table**

**2.1 Supplementary Table S1. Primer sequences used for qRT-PCR.**

| Gene | Species | Forward primer (5′–3′) | Reverse primer (5′–3′) |
| --- | --- | --- | --- |
| UNC93B1 | Human | TCTTCCATCTGAGCTTCGCC | GTCTTGTTGAAGCCGCTGAG |
| UNC93B1 | Mouse | CTTACCGGCCCACGGAA | CAAAGAGCACCTCAAAGCCACT |
| TGFβ | Human | ACCTGCCACAGATCCCCTAT | CTCCCGGCAAAAGGTAGGAG |
| CD206 | Human | GCCTCGTTGTTTTGCGTCTT | GAGAACAGCACCCGGAATGA |
| CD163 | Human | CTTGGGACTTGGACGATGCT | GCAGGACAATCCCACAAGGA |
| IL-10 | Human | TGAAAACAAGAGCAAGGCCG | GCCACCCTGATGTCTCAGTT |
| IL-6 | Human | TGAGGAGACTTGCCTGGTGAA | CAGCTCTGGCTTGTTCCTCAC |
| TGFβ | Mouse | GCCCGAAGCGGACTACTATG | ATAGATGGCGTTGTTGCGGT |
| CD206 | Mouse | ATGGATTGCCCTGAACAGCA | CTCGTCAGCACCCCAGTTAG |
| CD163 | Mouse | ATGCTTCCATCCAGTGCCTC | CACAAACCAAGAGTGCCGTG |
| IL-10 | Mouse | TGCAGTGTGTATTGAGTCTGCT | CGGAGAGAGGTACAAACGAGG |
| IL-6 | Mouse | AGACAAAGCCAGAGTCCTTCAG | TGTGACTCCAGCTTATCTCTTGG |
| CDK1 | Human | GGAAACCAGGAAGCCTAGCATC | GGATGATTCAGTGCCATTTTGCC |
| CCNA2 | Human | CTCTACACAGTCACGGGACAAAG | CTGTGGTGCTTTGAGGTAGGTC |
| GADPH | Human | TCCAAAATCAAGTGGGGCGA | AAATGAGCCCCAGCCTTCTC |
| ACTB | Human | CTCGCCTTTGCCGATCC | ATCCTTCTGACCCATGCCC |
| GADPH | Mouse | ACCCAGAAGACTGTGGATGG | CACATTGGGGGTAGGAACAC |
| ACTB | Mouse | TGTTACCAACTGGGACGACA | GGGGTGTTGAAGGTCTCAAA |

Supplementary Table S1. Primer sequences used for qRT-PCR.

Primer sequences used for quantitative real-time PCR validation of target genes in human and mouse cell lines. GAPDH or ACTB was used as the internal reference gene where appropriate.

**2.2 Supplementary Table S2. siRNA sequences used for UNC93B1 knockdown.**

| Target gene | Species | siRNA name | Sense sequence (5′–3′) | Antisense sequence (5′–3′) |
| --- | --- | --- | --- | --- |
| UNC93B1-760 | Human | Si-UNC93B1-1 | CAGCUGCCCAUGAUUUAUUTT | AAUAAAUCAUGGGCAGCUGTT |
| UNC93B1-614 | Human | Si-UNC93B1-2 | CGCAGAAGUACCAUGAGUATT | UACUCAUGGUACUUCUGCGTT |
| UNC93B1-1081 | Human | Si-UNC93B1-3 | CUCGUGCCUUUCUUUAUCUTT | AGAUAAAGAAAGGCACGAGTT |
| UNC93B1 | Human | Si-UNC93B1-Mix | sequence not disclosed by the supplier | sequence not disclosed by the supplier |
| Negative control | Human | Si-NC | UUCUCCGAACGUGUCACGUTT | ACGUGACACGUUCGGAGAATT |

Supplementary Table S2. siRNA sequences used for UNC93B1 knockdown.

The siRNA sequences targeting UNC93B1 and the corresponding negative control sequence used for transient knockdown experiments are listed. The most efficient siRNA sequence was selected for subsequent functional assays.

**2.3 Supplementary Table S3. Plasmid information used for UNC93B1 overexpression.**

| Target gene | Species | Vector | Tag | Insert | Application |
| --- | --- | --- | --- | --- | --- |
| UNC93B1 | Mouse | pCMV-SPORT6-UNC93B1 | Flag | Full-length mouse UNC93B1 clone | Overexpression |
| UNC93B1 | Mouse | pCMV-SPORT6 empty vector | None | None | Negative control for overexpression |

Supplementary Table S3. Plasmid information used for UNC93B1 overexpression.

For gain-of-function experiments, UNC93B1 overexpression was achieved by transfecting cells with the pCMV-SPORT6-UNC93B1 plasmid carrying a mouse UNC93B1 clone. Cells transfected with the corresponding pCMV-SPORT6 empty vector were used as vector controls.
